# Automatic and robust estimation of sex and chronological age from panoramic radiographs using a multi-task deep learning network: a study on a South Korean population

**DOI:** 10.1007/s00414-024-03204-4

**Published:** 2024-03-12

**Authors:** Se-Jin Park, Su Yang, Jun-Min Kim, Ju-Hee Kang, Jo-Eun Kim, Kyung-Hoe Huh, Sam-Sun Lee, Won-Jin Yi, Min-Suk Heo

**Affiliations:** 1 Department of Oral and Maxillofacial Radiology and Dental Research Institute, School of Dentistry, Seoul National University, Seoul, 03080 South Korea; 2 Department of Applied Bioengineering, Graduate School of Convergence Science and Technology, Seoul National University, Seoul, 03080 South Korea; 3 Department of Electronics and Information Engineering, Hansung University, Seoul, 03080 South Korea; 4 Department of Oral and Maxillofacial Radiology, School of Dentistry, Seoul National University, 101 Daehak-ro, Jongno-gu, Seoul, 03080 Korea

**Keywords:** Sex estimation, Age estimation, Panoramic radiographs, Deep learning, Multi-task learning

## Abstract

Sex and chronological age estimation are crucial in forensic investigations and research on individual identification. Although manual methods for sex and age estimation have been proposed, these processes are labor-intensive, time-consuming, and error-prone. The purpose of this study was to estimate sex and chronological age from panoramic radiographs automatically and robustly using a multi-task deep learning network (ForensicNet). ForensicNet consists of a backbone and both sex and age attention branches to learn anatomical context features of sex and chronological age from panoramic radiographs and enables the multi-task estimation of sex and chronological age in an end-to-end manner. To mitigate bias in the data distribution, our dataset was built using 13,200 images with 100 images for each sex and age range of 15–80 years. The ForensicNet with EfficientNet-B3 exhibited superior estimation performance with mean absolute errors of 2.93 ± 2.61 years and a coefficient of determination of 0.957 for chronological age, and achieved accuracy, specificity, and sensitivity values of 0.992, 0.993, and 0.990, respectively, for sex prediction. The network demonstrated that the proposed sex and age attention branches with a convolutional block attention module significantly improved the estimation performance for both sex and chronological age from panoramic radiographs of elderly patients. Consequently, we expect that ForensicNet will contribute to the automatic and accurate estimation of both sex and chronological age from panoramic radiographs.

**Supplementary Information:**

The online version contains supplementary material available at 10.1007/s00414-024-03204-4.

## Introduction

Sex and chronological age estimation are essential in forensic investigations and research for individual identification, which can help narrow down potential suspects. Given the well-preserved skull, panoramic radiographs can serve as a tool for identifying unidentified individuals in mass disasters and accidents [1, 2]. Various conventional methods have been employed for sex and chronological age estimation, including forensic deoxyribonucleic acid (DNA) analysis and morphological measurements of hard tissues such as teeth and bones. Forensic DNA analysis is a common method for sex and chronological age estimation that yields high accuracy and reliability [3]. However, forensic DNA analysis is time-consuming and labor-intensive; these problems can lead to challenges in terms of timeliness, particularly when there is a large caseload or when limited resources are available [4]. The hard tissues of the human body, such as teeth and bones, can preserve their shape and structure in hostile environments, making them suitable for sex and chronological age estimation in forensic applications [5, 6]. Recently, various dental-related parameters obtained from morphological measurements of anatomical structures, such as the maxillofacial bones, teeth, and frontal and paranasal sinuses, have been used in forensic dentistry for sex and age estimation [7–10]. These parameters can also be calculated from panoramic radiographs commonly used in the dental field to provide a broad view of the maxillofacial region as two-dimensional radiographic images [11].

Most dental age estimation methods involve radiographic assessment of teeth, which can provide information on skeletal maturity and is less affected by environmental factors [12]. Several methods are used to estimate dental age in children and adolescents using radiographic images. The Demirjian method was used to estimate chronological age by estimating the seven teeth on the left side of the mandible [13]. The Nolla method was used to evaluate the mineralization of permanent dentition in 10 stages. After assigning a value to each tooth, the sum of the values of maxillary and mandibular teeth was calculated and compared with the reference value [14]. The Cameriere method measures the ratio between the length of the projection of open apices and the length of the tooth axis major [15]. For dental age estimation in adults, the Kvaal method calculates the pulp-to-tooth ratio of six mandibular and maxillary teeth, including the maxillary central and lateral incisors, maxillary second premolars, mandibular lateral incisors, mandibular canines, first premolars. The coronal pulp cavity index was calculated as the correlation between the reduction in the coronal pulp cavity and chronological age, considering only the mandibular premolars and molars [6]. Recently, An et al. assessed age-related changes in dental development and the maturation of teeth and mandibular structures on panoramic radiographs. The results demonstrated changes in various radiographic parameters with increasing age [16].

Several studies have reported differences in tooth and bone size between males and females [8, 17]. These differences in the skeletal structure may serve as a preliminary reference for estimating sex. Recently, anatomical information on the maxillofacial and dental structures, such as mandibular angle; area of the mandibular foramen; the height of the symphysis in the mandible [18]; a volume of the maxillary, frontal, and paranasal sinuses [9]; crown dimension [19]; and pulp chamber volume [20], has been widely used for sex prediction. Although the aforementioned manual methods have been applied successfully to diverse populations, low reproducibility, and measurement bias remain limitations for clinical applications [21]. These manual methods include several steps, such as image preprocessing, manual segmentation, feature extraction, classification, and regression, and each step is labor-intensive, time-consuming, and error-prone [22]. Therefore, an automatic and accurate method for simultaneously estimating sex and chronological age using radiographs is required.

Recently, deep learning has been widely used for medical image analysis tasks, such as image segmentation, classification, detection, denoising, and synthesis [23–25]. Several studies have reported the use of deep learning-based methods for sex or age estimation from panoramic radiographs. Guo et al. [21] proposed a deep learning-based method to directly classify dental ages and compared it with a manual method on 10,257 panoramic radiographs of 4,579 males and 5,678 females aged between 5 and 24 years old. The results demonstrated that the deep learning-based method outperformed the manual method. Milošević et al. [26] investigated the potential use of deep learning in estimating chronological age based on panoramic radiographs. They built a dataset with 4,035 images from 2,368 males and 1,667 females aged 18–90 years. The performance of the age estimation model resulted in a mean absolute error (MAE) value of 3.96 ± 2.95. Bu et al. [27] investigated the potential use of a deep network in predicting sex based on panoramic radiographs of 10,703 patients (4,789 males and 5,914 females) aged 5–25 years. The accuracy of sex estimation using a convolutional neural network was higher for adults (90.97%) than for minors (82.64%). The deep learning-based methods simultaneously estimated sex and age from panoramic radiographs. Vila–Blanco et al. [28] proposed the use of DASNet to estimate sex and age based on 2,289 panoramic radiographs of subjects aged 4.5–89.2 years. The MAE value for age estimation was 2.84 ± 3.75 years, and the sex estimation accuracy was 85.4%. Fan et al. [29] estimated sex and age using DASE-Net for 15,195 panoramic radiographs aged 16–50; the MAE for age estimation was 2.61 years and the accuracy of sex estimation was 95.54%. These studies used datasets with insufficient or non-uniform sex and age distributions. In their datasets, over half of the total data were samples from individuals in their 20 and 30 s, with twice as many female samples as male samples. Zhang et al. [30] proposed a sex-prior guided Transformer-based model for chronological age estimation on 10,703 panoramic radiographs acquired from patients aged 5–25 and achieved an MAE of 0.80 for chronological age estimation. As far as we know, no previous study is based on a dataset with uniform sex and age distributions across the age range of 15–80 years.

The purpose of this study was to estimate sex and chronological age from panoramic radiographs automatically and robustly using a multi-task deep learning network (ForensicNet). To mitigate bias in the data distribution, our dataset was built using 13,200 images with 100 images for each sex and the age range from 15 to 80. Our main contributions are as follows: (1) A multi-task deep learning network was designed to automatically estimate the sex and chronological age simultaneously from panoramic radiographs in an end-to-end manner. (2) Using a convolutional block attention module (CBAM), a deep learning network was trained to learn the long-range relationships between anatomical structures for robust estimation of sex and chronological age from panoramic radiographs of elderly patients. In addition, the effectiveness of the CBAM was demonstrated by an experimental ablation study. (3) A weighted multi-task loss function was proposed to handle the imbalance of binary cross-entropy and MAE losses for estimating sex and chronological age.

## Materials and methods

### Data acquisition and preparation

Our dataset was built using 13,200 panoramic radiographs acquired from patients who underwent dental imaging at the Seoul National University Dental Hospital between 2017 and 2021 in South Korea. This study was approved by the Institutional Review Board of Seoul National University Dental Hospital (ERI23025). The ethics committee approved the waiver of informed consent because this was a retrospective study. The study was performed following the Declaration of Helsinki. Panoramic radiographs were acquired using OP-100 (Instrumentarium Dental, Tuusula, Finland), Rayscan alpha-P (Ray, Seoul, South Korea), and Rayscan alpha-OCL (Ray, Seoul, South Korea) under conditions of tube energy of 73 kVp and tube current of 10 mA.

**Fig. 1:**
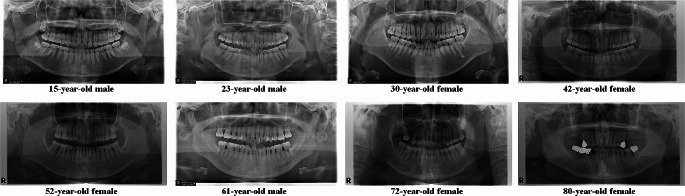
Examples of panoramic radiographs of males or females aged 15–80 years

The collected panoramic radiographs were unfiltered real-world data. We excluded only low-quality images caused by artifacts (the patient’s earrings, removable prosthesis, etc.), inadequate anatomical coverage, patient positioning errors, and pre-and post-processing errors (noise, enhancement errors, abnormal density, and contrast) [31]. Representative samples of patients aged 15–80 years from our dataset are shown in Fig. 1. Our dataset included panoramic radiographs acquired from patients with alterations, dental implants, caries, bridges, fillings, retainers, missing teeth, or crowns. However, the exclusion criteria were as follows: edentulous patients, patients undergoing orthodontic treatment, patients undergoing orthognathic surgery, maxillofacial reconstruction patients, and patients with large intraosseous lesions.

Each panoramic radiograph was labeled with the specific sex and chronological age of the patient. Our dataset has the same distribution of sex and chronological age, with approximately equal numbers of images for each sex and age group. The datasets were randomly separated into training, validation, and test sets, where each set consisted of the same distribution of sex (male and female) and chronological age (15–80 years old). The splitting ratio was 3:1:1, and each set contained 7920, 2640, and 2640 images [32]. The dataset consists of high-resolution 8-bit panoramic radiographs. The heights of the panoramic radiographs ranged from 976 to 1468 pixels, while the widths ranged from 1976 to 2988 pixels. For the network training, the images were resized to 480 $\times$ 960 pixels.

The minimum sample size was estimated to detect significant differences in accuracy between ForensicNet and the other networks when both assessed the same subjects (panoramic radiographs). Sample size calculation was designed to capture a mean accuracy difference of 0.05 and a standard deviation of 0.10 between the ForensicNet and other networks. Based on an effect size of 0.25, a significance level of 0.05, and a statistical power of 0.95, a sample size of 305 was obtained by G* Power (Windows 10, version 3.1.9.7; Universität Düsseldorf, Germany). The dataset of panoramic radiographs was split into 7920, 2640, and 2640 images for the training, validation, and test sets, respectively.

### Proposed multi-task deep learning network (ForensicNet)

**Fig. 2:**
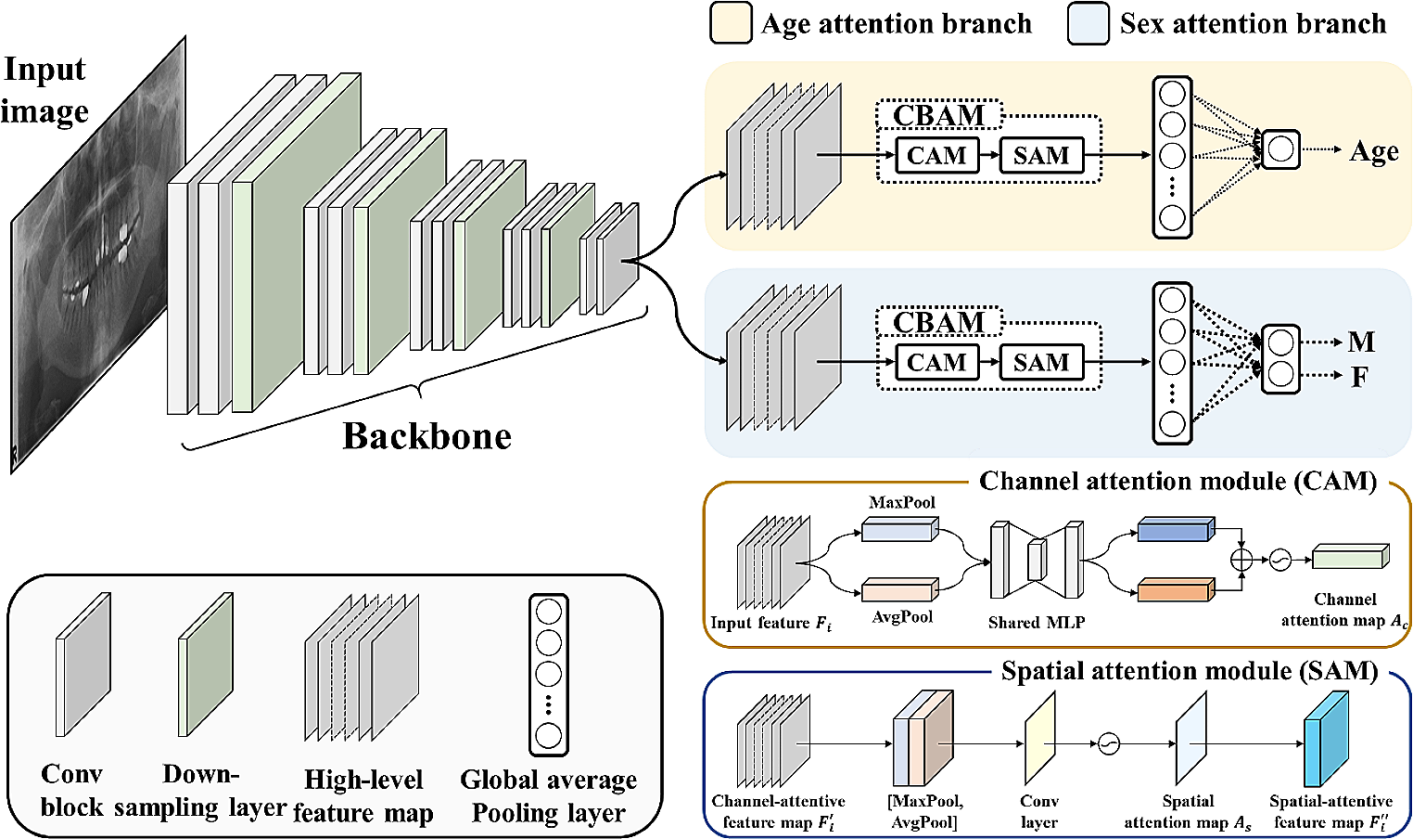
Overview of the proposed multi-task deep learning network (ForensicNet). ForensicNet consists of a backbone with age and sex attention branches. Each attention branch has a convolutional block attention module (CBAM) composed of channel and spatial attention modules. ForensicNet takes panoramic radiographs as inputs and simultaneously estimates sex and chronological age by each attention branch

The architecture of the proposed network, ForensicNet, consisted of a backbone, sex, and age attention branches (Fig. 2). Popular feature extraction networks such as VGG16 [33], MobileNet v2 [34], ResNet101 [35], DenseNet121 [36], Vision Transformer [37], Swin Transformer [38], Encoder of TransUNet (TransNet) [39], and EfficientNet-B3 [40] were used as backbones in ForensicNet.

VGG16 consists of 16 layers, including 13 convolutional layers with ReLU activation, 5 max-pooling layers, and 3 fully connected layers. VGG16 contains approximately 15.1 million trainable parameters [33]. MobileNet v2 is designed to implement the inference of deep networks with low computing power, such as mobile devices [34]. To design a lightweight model, MobileNet v2 uses depth-wise separable convolutions instead of standard convolutions. MobileNet v2 has approximately 4.7 million trainable parameters. A residual neural network, also called ResNet, adopts a residual learning method that employs the addition of a skip connection between layers [35]. This skip connection is an element-wise addition between the input and output of the residual block, without additional parameters or computational complexity. ResNet101 contained 48.8 million trainable parameters. The densely connected network DenseNet121 uses a cross-layer connection approach in each layer to solve the problem of the vanishing gradient. In the DenseNet121 architecture, the feature maps of each previous layer are used as inputs for all subsequent layers. DenseNet121 contains approximately 8.6 million trainable parameters [36]. Vision Transformer adapts the original Transformer architecture for use in computer vision [37]. It takes an input image by dividing it into non-overlapping patches and generating the linear embedding from these patches based on the linear projection. To include the location information of each patch, positional encodings are appended to this linear embedding. Subsequently, these embedding vectors are fed into a Transformer encoder. Vision Transformer contains approximately 87.0 million trainable parameters [37]. Swin Transformer is a type of Transformer architecture that has been specifically designed for computer vision tasks [38]. Swin Transformer applies shifted local windows in an image across different levels of detail, allowing the model to capture both local details and global context. Swin Transformer contains approximately 89.8 million trainable parameters [38]. TransNet is the encoder of TransUNet which combines the advantages of Transformer and convolutional neural networks (CNN) to improve segmentation performance by capturing both global and local features [39]. In TransNet, ResNet50 is used as a CNN-based encoder to extract high-level features. Then, high-level features are fed to the Transformer with self-attention layers to capture global contextual relationships. TransNet contains approximately 31.5 million trainable parameters [39]. EfficientNet is a state-of-the-art network that significantly outperforms other popular networks in classification tasks with fewer parameters and high model efficiency. EfficientNet employs a compound scaling method to efficiently adjust the width, depth, and resolution of a deep network. EfficientNet-B3 contains approximately 14.3 million trainable parameters [40].

On panoramic radiographs, anatomical structures are typically observed in different sizes and shape variations according to the sex and chronological age of the patients. To learn these features, a deep network must cover different scales of receptive fields to capture long-range relationships between anatomical structures. In this study, a CBAM [41] was embedded before each output layer in the sex and age attention branches of the proposed ForensicNet. The CBAM contained two submodules for channel and spatial attention (Fig. 2). An input feature map ${F}_{i}\in {\mathbb{R}}^{C\times H\times W}$ are fed to the channel attention module (CAM) to obtain a 1D channel attention map ${A}_{c}\in {\mathbb{R}}^{C\times 1\times 1}$ as follows:

1 $${A}_{c}=\sigma \left(\text{M}\text{L}\text{P}\right(\text{M}\text{a}\text{x}\text{P}\text{o}\text{o}\text{l}\left({F}_{i}\right)\left)\right)+\sigma \left(\text{M}\text{L}\text{P}\right(\text{A}\text{v}\text{g}\text{P}\text{o}\text{o}\text{l}\left({F}_{i}\right)\left)\right)$$,

where $C$, $H$, and $W$ indicate channels, height, and width of a feature map, respectively. $\sigma$, MLP, MaxPool, and AvgPool denote the Sigmoid activation function, shared multi-layer perceptron layers, a global max-pooling layer, and a global average-pooling layer, respectively. Then, a channel-attentive feature map ${F}_{i}^{{\prime }}\in {\mathbb{R}}^{C\times H\times W}$ is acquired by:

2 $${F}_{i}^{{\prime }}={A}_{c}\otimes {F}_{i}$$,

where $\otimes$ denotes element-wise multiplication. To obtain a spatial attention feature map ${A}_{s}\in {\mathbb{R}}^{1\times H\times W}$, a channel-attentive feature map ${F}_{i}^{{\prime }}$ is fed to the spatial attention module (SAM) as follows:

3 $${A}_{s}=\sigma \left({s}^{7\times 7}\left(\left[\text{M}\text{a}\text{x}\text{P}\text{o}\text{o}\text{l}\left({F}_{i}^{{\prime }}\right); \text{A}\text{v}\text{g}\text{P}\text{o}\text{o}\text{l}\left({F}_{i}^{{\prime }}\right)\right]\right)\right)$$,

where $\sigma$, ${s}^{7\times 7}$, MaxPool, and AvgPool denote the Sigmoid activation function, a $7\times 7$ convolution layer, a 2D max-pooling layer, and a 2D average-pooling layer, respectively. $\left[\bullet \right]$ indicates channel-wise concatenation operation. Then, a spatial-attentive feature map ${F}_{i}^{{\prime }{\prime }}\in {\mathbb{R}}^{C\times H\times W}$ is obtained by:

4 $${F}_{i}^{{\prime }{\prime }}={A}_{s}\otimes {F}_{i}^{{\prime }}$$,

where $\otimes$ denotes element-wise multiplication. Finally, a spatial-attentive feature map ${F}_{i}^{{\prime }{\prime }}$of CBAM combined with spatial and channel attention were fed to a global average pooling layer. CBAM can promote deep networks to focus on semantic information and effectively refine intermediate features.

To output multi-task classes for both sex and chronological age estimation in an end-to-end manner, sex and age attention branches were designed, where each branch comprised a CBAM, a global average pooling layer, and an output layer (Fig. 2). In the age attention branch, high-level feature maps from the backbone were fed to the CBAM to extract channel and spatial attentive feature maps. The channel and spatial attentive feature maps were then reduced to a one-dimensional vector using a global average pooling layer, and the vector was fed to an output layer with a linear activation function to estimate a continuous age value. The sex attention branch had the same structure as the attention branch, except for the activation function of the output layer, where sigmoid activation was used to classify a categorical sex value, such as male or female.

### Weighted multi-task loss function

For network training, a weighted multi-task loss (WML) function combined with MAE and binary cross-entropy (BCE) was proposed. The MAE measures the mean of the absolute difference between the ground truth and the estimated chronological age. The MAE is defined as

5 $$MAE\left(y,\widehat{y}\right)=\frac{{\sum }_{i=1}^{N}\left|{y}_{i}-{\widehat{y}}_{i}\right|}{N}$$,

where $y$ and $\widehat{y}$ are the ground truth and estimated chronological ages, respectively. The $N$ is the number of panoramic radiographs. The BCE measures the average probability error between the ground truth and the estimated sex. The BCE is defined as follows:

6 $$BCE\left(p, \widehat{p}\right)=-{\sum }_{i=1}^{N}{p}_{i}{log}\left({\widehat{p}}_{i}\right)$$,

where $p$ and $\widehat{p}$ are the ground truth and estimated sex, respectively. $N$ is the number of panoramic radiographs. The MAE was more difficult to minimize than the BCE for multi-task learning. Therefore, asymmetric weights $\alpha$ and $\beta$ for MAE and BCE were set in WML, respectively. Finally, the WML is defined as

7 $$WML={\upalpha }MAE\left(y,\widehat{y}\right)+ \beta BCE\left(p, \widehat{p}\right),$$

where $\alpha$ and $\beta$ are weight constants for MSE and BCE, respectively, and the $\beta$is calculated as $\left(1-\alpha \right)$. Empirically, $\alpha$ and $\beta$ were set to 0.7 and 0.3 (Table 1), respectively.

**Table 1:** Performance comparison of sex and chronological age estimation by changing backbones in ForensicNet

Backbones	Chronological age				Sex		
	MAE (years)	MD (years)	R^2^	ACC		SPE	SEN
VGG16	3.43 ± 3.07^*^	17.03	0.941	0.977		0.975	0.978
MobileNet v2	3.07 ± 2.70	15.80	0.953	0.987		0.982	0.993
ResNet101	3.03 ± 2.67	13.09	0.954	0.985		0.996	0.975
DenseNet121	3.00 ± 2.68	15.82	0.955	0.988		0.981	0.995
Vision Transformer	3.56 ± 3.25^†^	23.94	0.933	0.967		0.954	0.979
Swin Transformer	3.39 ± 3.15^‡^	21.87	0.938	0.979		0.991	0.968
TransNet	3.99 ± 3.44^**^	22.63	0.920	0.912^††^		0.960	0.862
EfficientNet-B3	2.93 ± 2.61	16.31	0.957	0.992		0.993	0.990

MAE, mean absolute error; MD, maximum deviation; R^2^, coefficient of determination; ACC, accuracy; SPE, specificity; SEN, sensitivity. ^*^Significant difference for MAE between VGG16 and the others except for Vision Transformer and Swin Transformer (*p*-value < 0.05). ^†^Significant difference for MAE between Vision Transformer and the others except for VGG16 and Swin Transformer (*p*-value < 0.05). ^‡^Significant difference for MAE between Swin Transformer and the others except for VGG16 and Swin Transformer (*p*-value < 0.05). ^**^Significant difference for MAE between TransNet and the others (*p*-value < 0.05). ^††^Significant difference in sex estimation performance between TransNet and the others except for Swin Transformer (*p*-value < 0.05)

### Training environment

The deep networks were trained for 200 epochs with a mini-batch size of 16. Data augmentation was performed with rotation (ranging from − 10° to 10°) and width and height shifts (ranging from − 10 to 10% of the image size) in the horizontal and vertical axes. Adam optimizer was used with ${\beta }_{0}=0.9$ and ${\beta }_{1}=0.999$, and a learning rate was initially set to 10^−3^, which was reduced by half up to 10^−6^ when the validation loss saturated for 25 epochs. The deep networks were implemented using Python3 based on Keras with a TensorFlow backend, using an NVIDIA TITAN RTX GPU of 24GB.

### Evaluation metrics

To evaluate the estimation performance for sex and chronological age, the MAE, coefficient of determination (R^2^), maximum deviation (MD), successful estimation rate (SER), sensitivity (SEN), specificity (SPE), and accuracy (ACC) were used. The MAE is the mean of the absolute difference between the estimated and actual ages of a sample. R^2^ is a statistical measure of the fit of a regression model (measures the variations in the data explained by the model). Maximum Deviation (MD) is the highest deviation of the absolute difference between the estimated and actual ages, compared to their mean. SER is the percentage of successfully estimated ages in the ranges of 1-, 3-, 5-, 8-, and 10-year errors, and SEN is a metric that evaluates the ability of a model to estimate the true positives of each available category of sex. SPE is a metric that evaluates the ability of a model to estimate the true negatives of each available category of sex. ACC is the ratio of the number of correct sex estimations to the total number of input samples.

The impact of dataset size on the estimation of sex and chronological age was also evaluated. The training sets were expanded to include 2640, 5260, and 7920 images, respectively, while the validation and test sets were fixed. An analysis of variance test was performed to compare the estimation performances between the backbones in ForensicNet (PSS Statistics for Windows 10, Version 26.0; IBM, Armonk, New York, USA), and the statistical significance level (*p*-value) was set to 0.05.

To interpret the decision-making processes of a deep network, gradient-weighted class activation mapping (Grad-CAM) was used [42]. Grad-CAM is used to visualize the heatmap of the regions that the deep network focuses on when making an estimation. This method calculates the gradients of the target (here, an output layer to estimate sex and chronological age) and plugs them into a previous convolutional layer to provide a heatmap of the regions that contribute the most to the output decision.

## Results

**Fig. 3:**
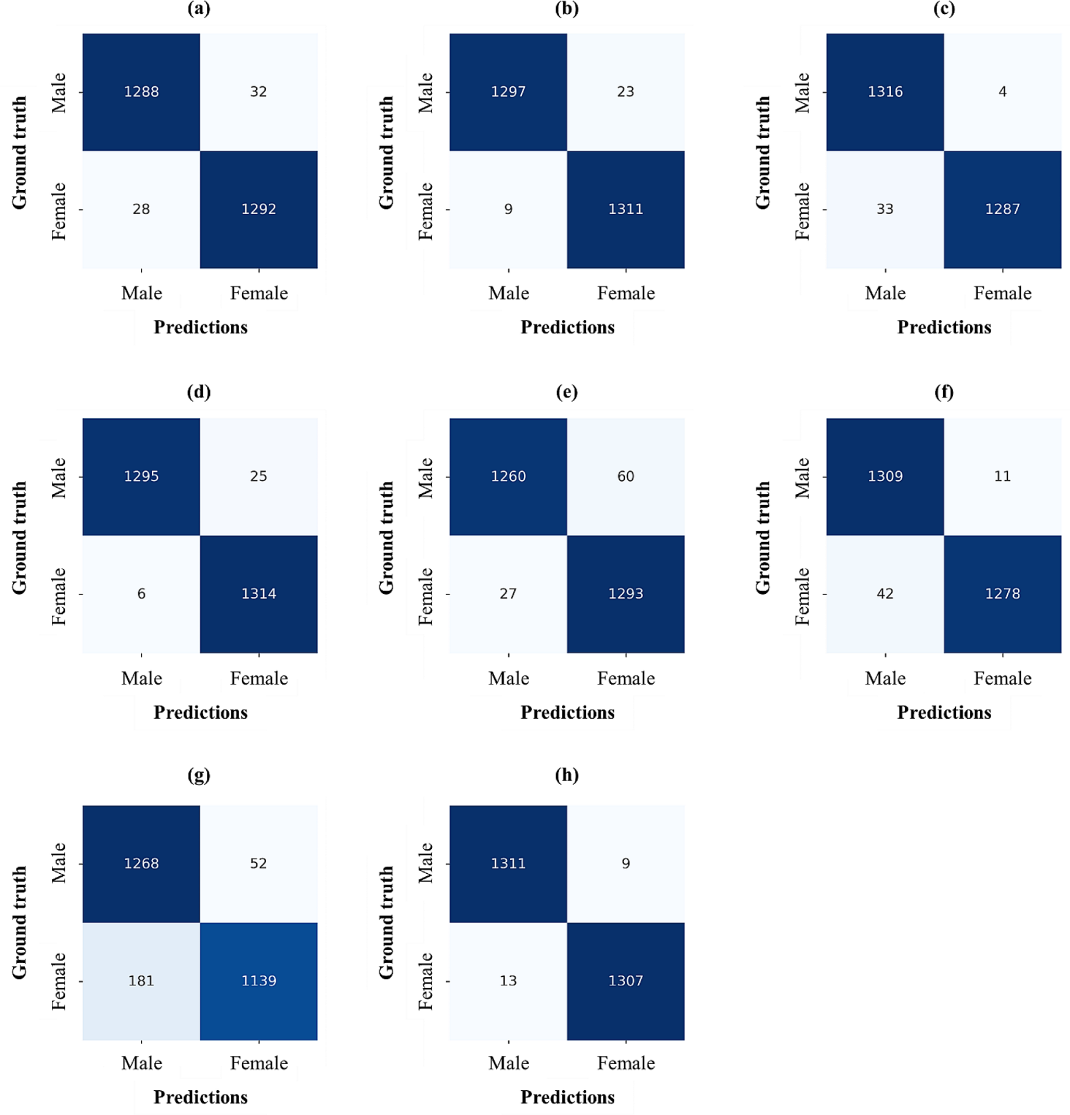
Confusion matrices for sex estimation from different backbones. (a)–(h) Results of VGG16, MobileNet v2, ResNet101, DenseNet121, Vision Transformer, Swin Transformer, TransNet, and EfficientNet-B3, respectively

The performances of backbones such as VGG16, MobileNet v2, ResNet101, DenseNet121, Vision Transformer, Swin Transformer, TransNet, and EfficientNet-B3 used in ForensicNet were compared. To ensure a fair comparison, all the deep networks were run in the same computing environment and with the same data augmentations used in our comparative experiments. As shown in Table 2, all deep networks achieved high estimation performance for sex and chronological age from the panoramic radiographs. In estimating sex and chronological age, EfficientNet-B3 outperformed the other backbones for most evaluation metrics, particularly in the estimation performance of chronological age. From the quantitative results of the sex estimation, EfficientNet-B3 achieved ACC, SPE, and SEN values of 0.992, 0.993, and 0.990, respectively (Table 2). Compared with the second-highest results from DenseNet121, the ACC and SPE of EfficientNet-B3 improved by 0.004 and 0.012, respectively. Significant differences were observed in the MAE between EfficientNet-B3 and the other backbones including VGG16, Vision Transformer, Swin Transformer, and TransNet (*p*-value < 0.05), whereas no significant differences were observed in the sex estimation performance except for those of TransNet (Table 2). Figure 3 illustrates the confusion matrices for the sex estimation performance of all backbones.

**Table 2:** Successful estimation rate of different backbones in ForensicNet for chronological age estimation. Results indicate the percentage of successfully estimated ages in the ranges of 1-, 3-, 5-, 8-, and 10-year errors

Backbones	< 1.0	< 3.0	< 5.0	< 8.0	< 10.0
VGG16	24.16	55.08	75.27	91.21	95.95
MobileNet v2	24.39	59.43	81.02	93.97	97.57
ResNet101	25.83	60.15	80.11	94.28	97.27
DenseNet121	26.66	60.19	80.23	93.67	97.58
Vision Transformer	22.23	54.92	73.82	89.96	95.60
Swin Transformer	25.03	57.00	75.68	90.96	96.02
TransNet	19.31	49.28	68.82	86.85	92.99
EfficientNet-B3	26.78	61.74	81.55	94.09	97.99

**Fig. 4:**
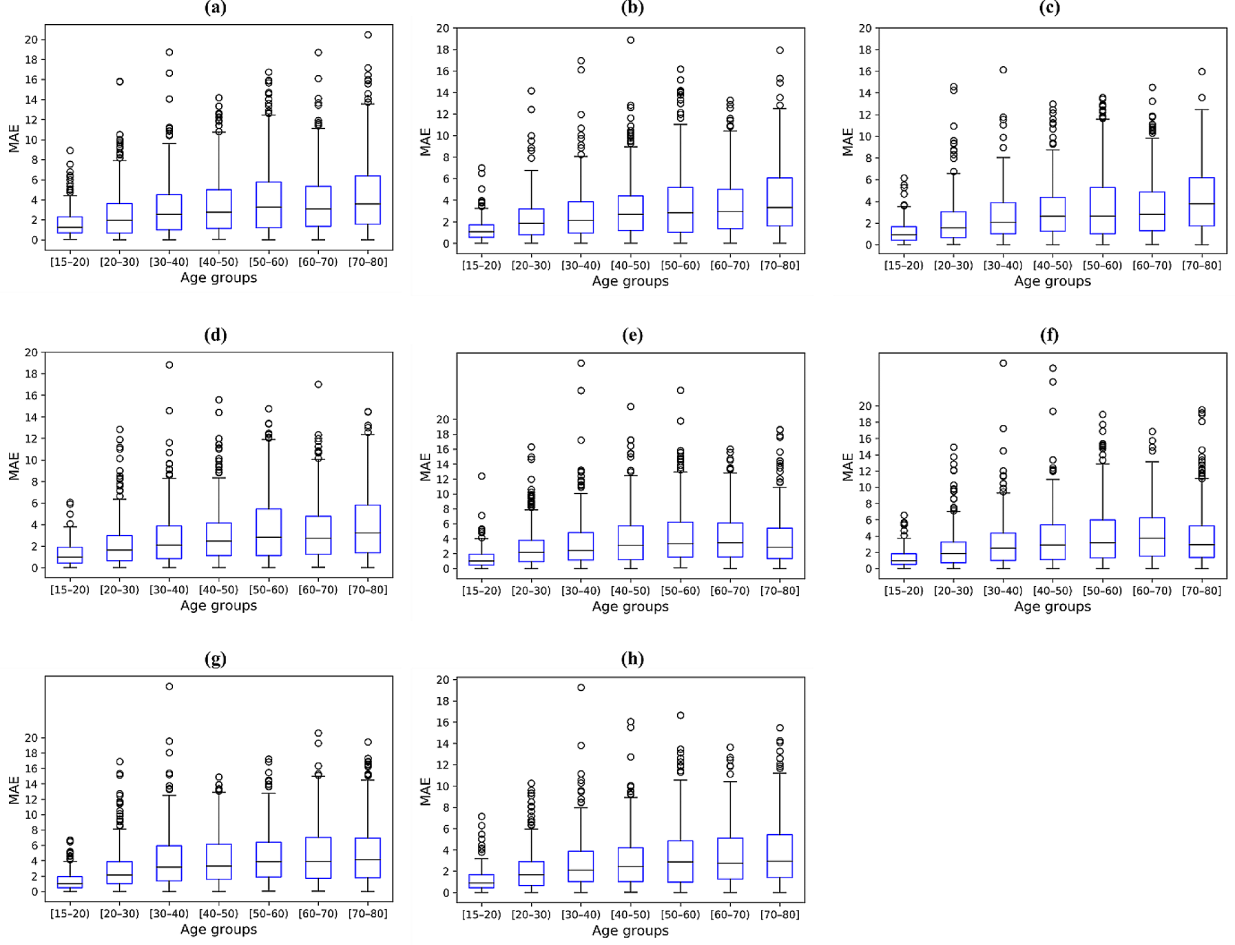
Box plots for estimation performance of chronological age from different backbones on each age group. Each blue box contains the first and third quartiles of accuracy. Medians are located inside the blue boxes as black lines, with the minimum and maximum values visualized as vertical lines. Black circles are outliers. (a)–(h) Results of VGG16, MobileNet v2, ResNet101, DenseNet121, Vision Transformer, Swin Transformer, TransNet, and EfficientNet-B3, respectively

**Fig. 5:**
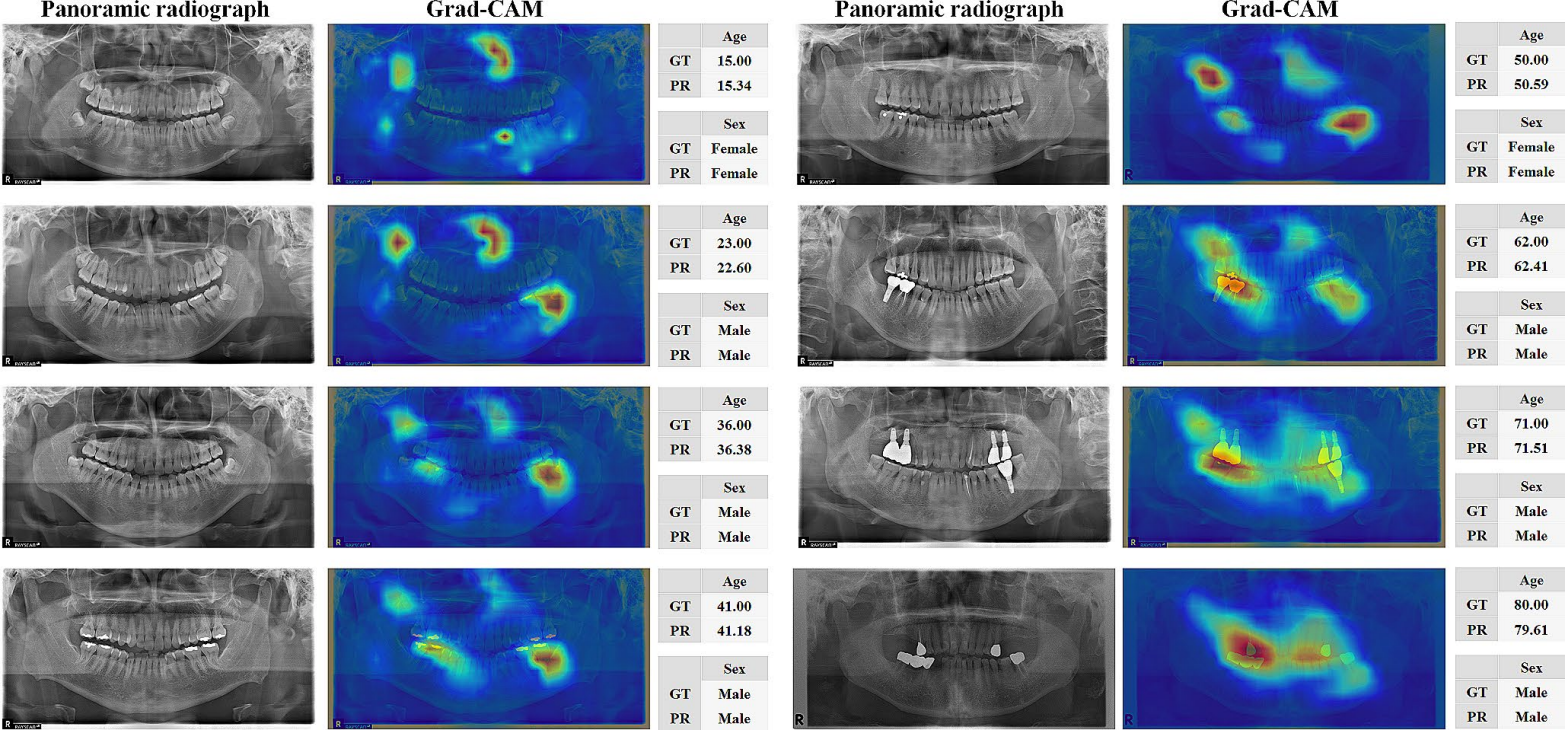
Representative estimation results and corresponding Grad-CAM generated by EfficientNet-B3. GT and PR are the ground truth and estimation results, respectively

**Fig. 6:**
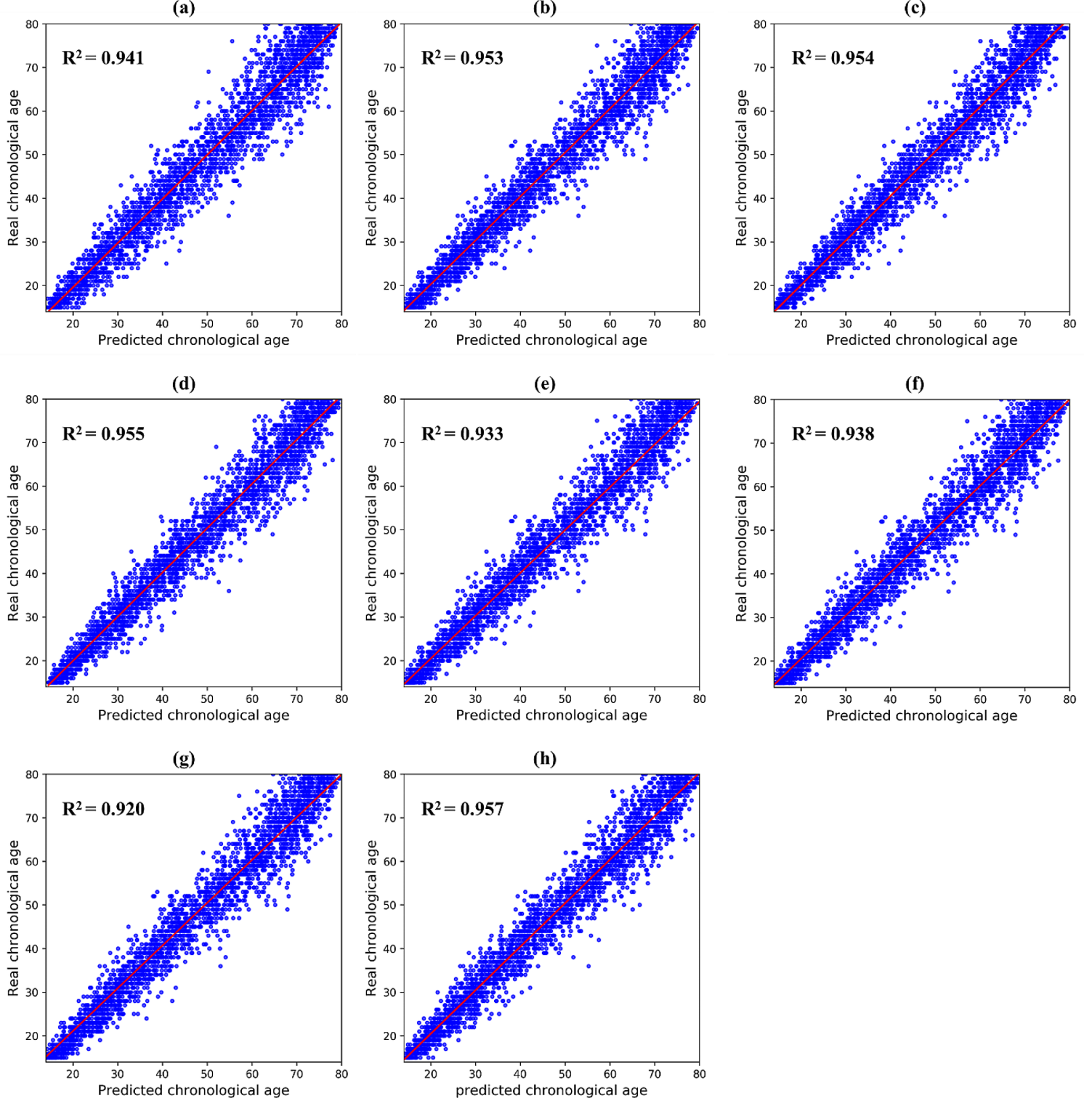
Linear regression plots for estimation performance of chronological age from different backbones. Blue dots are observations between ground truth and estimated ages, and the red line denotes a linear regression line. R^2^ is a measure of the goodness of fit of a backbone. (a)–(h) Results of VGG16, MobileNet v2, ResNet101, DenseNet121, Vision Transformer, Swin Transformer, TransNet, and EfficientNet-B3, respectively

**Fig. 7:**
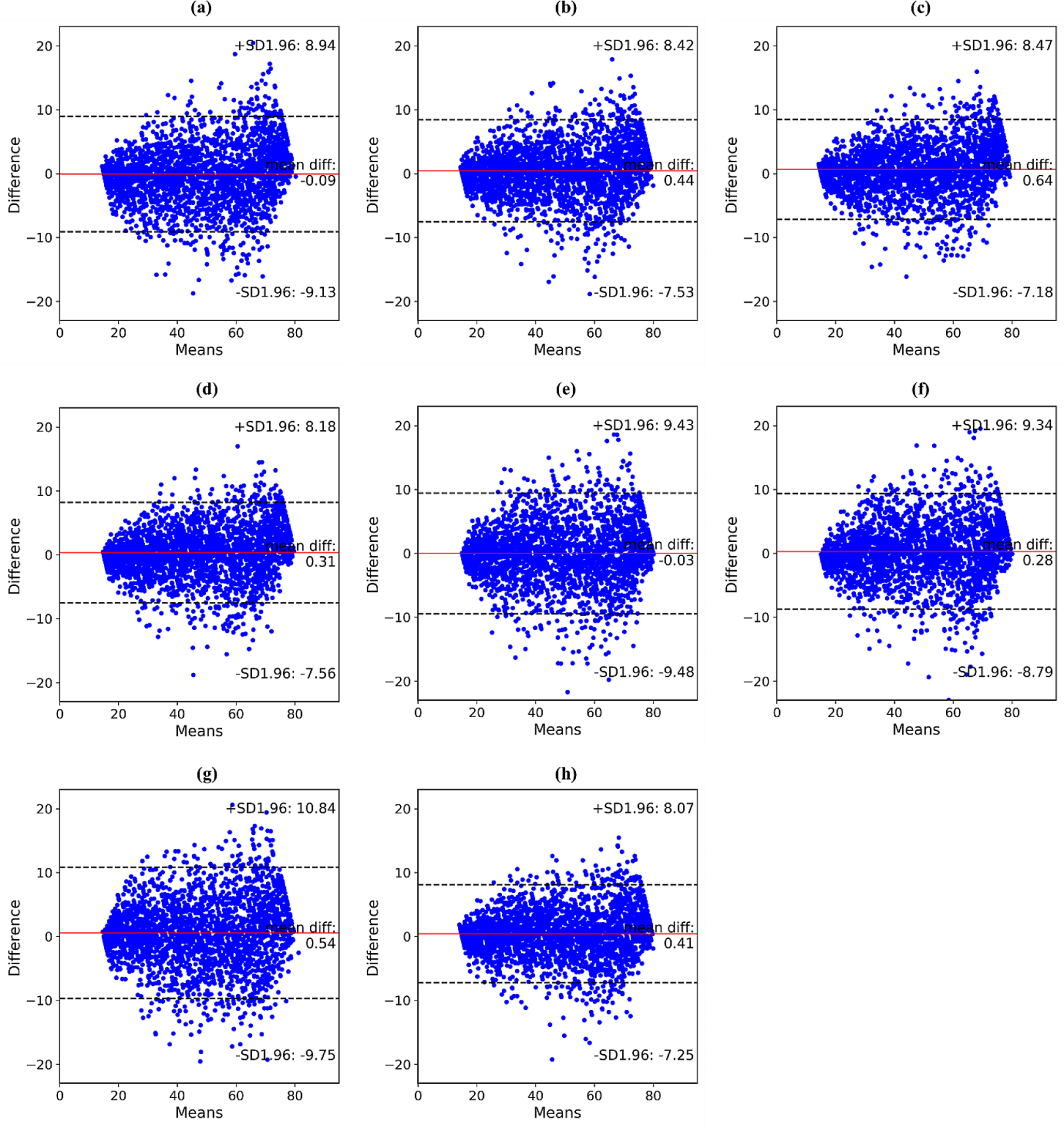
Bland–Altman plots for estimation performance of chronological age by backbones. Blue dots denote the differences between ground truth and estimated ages, the red line presents a mean difference, and black dash lines are 95% limits of agreement. (a)–(h) Results of VGG16, MobileNet v2, ResNet101, DenseNet121, Vision Transformer, Swin Transformer, TransNet, and EfficientNet-B3, respectively

In chronological age estimation, EfficientNet-B3 achieved better results with MAE of 2.93 ± 2.61, MD of 16.13, R^2^ of 0.957, and SERs of 26.78, 61.74, 81.55, 94.09, and 97.99% than those of the other backbones (Tables 2 and 3). When comparing the estimation performances for each chronological age group, all deep networks exhibited a gradual increase in age estimation errors (Table 4). In addition, the median errors in age estimation gradually increased, as shown in Fig. 4. EfficientNet-B3 obtained an estimation performance comparable to that of the other backbones from panoramic radiographs acquired from patients younger than 50 years, whereas it achieved superior performance improvements in those obtained from patients older than 50 years. Figure 5 shows the representative results with the ground truth and the estimated results for both sex and chronological age from EfficientNet-B3. Figures 6 and 7 show the linear regression and Bland–Altman plots for chronological age estimation, respectively.

**Table 3:** Performance comparison of different backbones in ForensicNet on each chronological age group. Results are evaluated using MAE (years) and standard deviation

Backbones	[15–20)	[20–30)	[30–40)	[40–50)	[50–60)	[60–70)	[70–80]
VGG16	1.71 ± 1.58	2.54 ± 2.42	3.21 ± 2.80	3.48 ± 2.95	3.99 ± 3.43	3.73 ± 3.10	4.41 ± 3.53
MobileNet v2	1.31 ± 1.07	2.24 ± 1.91	2.69 ± 2.31	3.16 ± 2.61	3.59 ± 3.14	3.51 ± 3.72	4.04 ± 3.13
ResNet101	1.19 ± 1.06	2.13 ± 2.06	2.66 ± 2.22	3.09 ± 2.43	3.50 ± 2.43	3.37 ± 2.71	4.24 ± 3.11
DenseNet121	1.27 ± 1.12	2.12 ± 1.99	2.69 ± 2.41	3.03 ± 2.55	3.64 ± 3.05	3.39 ± 2.75	3.92 ± 3.00
Vision Transformer	1.39 ± 1.44	2.75 ± 2.51	3.40 ± 3.25	3.96 ± 3.46	4.35 ± 3.63	4.17 ± 3.22	3.81 ± 3.29
Swin Transformer	1.29 ± 1.10	2.38 ± 2.33	3.14 ± 2.86	3.69 ± 3.29	4.12 ± 3.58	4.28 ± 3.31	3.74 ± 3.28
TransNet	1.42 ± 1.34	2.85 ± 2.61	4.08 ± 3.50	4.08 ± 3.18	4.53 ± 3.34	4.72 ± 3.68	4.91 ± 3.86
EfficientNet-B3	1.23 ± 1.12	2.08 ± 1.84	2.68 ± 2.31	2.96 ± 2.49	3.48 ± 3.00	3.45 ± 2.80	3.69 ± 2.93

**Table 4:** Ablation study for CBAM in ForensicNet

Models	Chronological age			Sex		
	MAE (years)	MD (years)	R^2^	ACC	SPE	SEN
EfficientNet-B3 without CBAM	3.07 ± 2.67^*^	18.89	0.952	0.908^†^	0.995	0.822
EfficientNet-B3 with CBAM	2.93 ± 2.61	16.31	0.957	0.992	0.993	0.990

MAE, mean absolute error; R^2^, coefficient of determination; ACC, accuracy; SPE, specificity; SEN, sensitivity. ^*^Significant difference in MAE between models with and without CBAM (*p*-value < 0.05); ^†^Significant difference in sex estimation performance between models with and without CBAM (*p*-value < 0.05)

Ablation studies were performed to demonstrate the effectiveness of CBAM in ForensicNet (Table 5). For sex estimation, ForensicNet without CBAM obtained lower ACC and SEN of 0.908 and 0.882, respectively, than with CBAM of 0.992 and 0.990. Furthermore, the estimation performance of chronological age was improved from the MAE of 3.07 ± 2.67 to 2.93 ± 2.61 by embedding CBAM in ForensicNet. As shown in Table 5, significant differences were observed in both MAE and sex estimation performance between the models with and without CBAM (*p*-value < 0.05). In addition, the models with CBAM achieved performance improvements in panoramic radiographs obtained from patients older than 50 years (Table 6).

**Table 5:** Ablation study for CBAM in ForensicNet on each chronological age group. Results are evaluated using MAE (years) and standard deviation

Models	[15–20)	[20–30)	[30–40)	[40–50)	[50–60)	[60–70)	[70–80]
EfficientNet-B3 without CBAM	1.22 ± 1.04	2.08 ± 1.92	2.88 ± 2.43	3.24 ± 2.52	3.65 ± 2.97	3.55 ± 2.89	3.92 ± 2.94
EfficientNet-B3 with CBAM	1.23 ± 1.12	2.08 ± 1.84	2.68 ± 2.31	2.96 ± 2.49	3.48 ± 3.00	3.45 ± 2.80	3.69 ± 2.93

**Table 6:** Ablation study for $\alpha$ and $\beta$ weights of the weighted multi-task loss in EfficientNet-B3 with CBAM

Loss weights		Chronological age			Sex		
$\alpha$	$\beta$	MAE (years)	MD (years)	R^2^	ACC	SPE	SEN
0.1	0.9	2.95 ± 2.63	16.98	0.956	0.991	0.990	0.992
0.2	0.8	3.00 ± 2.64	14.15	0.953	0.990	0.987	0.992
0.3	0.7	2.99 ± 2.67	15.85	0.954	0.989	0.992	0.987
0.4	0.6	2.98 ± 2.70	19.36	0.954	0.989	0.983	0.994
0.5	0.5	2.97 ± 2.69	19.35	0.954	0.994	0.997	0.990
0.6	0.4	2.99 ± 2.64	17.27	0.954	0.989	0.994	0.984
**0.7**	**0.3**	2.93 ± 2.61	16.31	0.957	0.992	0.993	0.990
0.8	0.2	2.97 ± 2.67	14.91	0.953	0.979	0.969	0.989
0.9	0.1	2.97 ± 2.68	17.41	0.954	0.984	0.981	0.988

To find the optimal weights of WML in ForensicNet, we conducted ablation studies to compare the performance of different weight values between $\alpha$ and $\beta$. Table 1 shows the quantitative results from ForensicNet according to the different weight values of $\alpha$ and $\beta$ in WML. When $\alpha$ and $\beta$ were set as 0.7 and 0.3 respectively, ForensicNet achieved the superior estimation performance for sex and chronological age. In addition, the ForensicNet with the optimal weights of $\alpha$ and $\beta$ achieved performance improvements in panoramic radiographs obtained from patients older than 40 years (Table 7).

**Table 7:** Ablation study for $\alpha$ and $\beta$ weights of the weighted multi-task loss in EfficientNet-B3 with CBAM on each chronological age group. Results are evaluated using MAE (years) and standard deviation

Loss weights		[15–20)	[20–30)	[30–40)	[40–50)	[50–60)	[60–70)	[70–80]
$\alpha$	$\beta$							
0.1	0.9	1.14 ± 1.06	2.12 ± 1.85	2.69 ± 2.32	3.10 ± 2.52	3.57 ± 3.12	3.45 ± 2.72	3.59 ± 2.88
0.2	0.8	1.17 ± 1.10	2.19 ± 1.93	2.70 ± 2.24	2.97 ± 2.40	3.44 ± 2.98	3.47 ± 2.76	4.04 ± 3.07
0.3	0.7	1.12 ± 1.01	2.14 ± 2.02	2.69 ± 2.33	3.07 ± 2.63	3.70 ± 3.07	3.45 ± 2.75	3.75 ± 2.86
0.4	0.6	1.19 ± 1.10	2.10 ± 1.97	2.63 ± 2.48	3.07 ± 2.56	3.70 ± 3.06	3.56 ± 2.88	3.61 ± 2.92
0.5	0.5	1.13 ± 1.14	2.15 ± 1.92	2.73 ± 2.38	3.08 ± 2.56	3.59 ± 3.04	3.54 ± 2.77	3.67 ± 2.86
0.6	0.4	1.34 ± 1.20	2.15 ± 1.90	2.68 ± 2.32	3.12 ± 2.62	3.61 ± 3.05	3.40 ± 2.74	3.59 ± 2.89
**0.7**	**0.3**	1.23 ± 1.12	2.08 ± 1.84	2.68 ± 2.31	2.96 ± 2.49	3.48 ± 3.00	3.45 ± 2.80	3.69 ± 2.93
0.8	0.2	1.13 ± 1.04	2.18 ± 1.92	2.68 ± 2.44	3.03 ± 2.58	3.46 ± 2.93	3.50 ± 2.78	3.82 ± 3.03
0.9	0.1	1.18 ± 1.11	2.19 ± 1.95	2.67 ± 2.36	3.09 ± 2.55	3.60 ± 3.10	3.46 ± 2.86	3.72 ± 2.94

In Table 8, the impact of dataset size in ForensicNet with EfficientNet-B3 was validated. The results exhibited better estimation performance for sex and chronological age by further increasing the training dataset. Even when only approximately a quarter of the total dataset was used for network training, the estimation performance for sex and chronological age achieved an ACC of 0.963 and an MAE of 3.63 ± 2.98, respectively. By increasing the size of the training dataset, the estimation performance for sex and chronological age gradually improved, achieving an ACC of 0.992 and an MAE of 2.93 ± 2.61, respectively. Significant differences were observed in both MAE and sex estimation performance between the data sizes of 7920 and 2640 images (*p*-value < 0.05), whereas no significant differences were observed for sex estimation performance between the data sizes of 7920 and 5280 images (Table 8).

**Table 8:** Impacts of dataset size in ForensicNet with EfficientNet-B3. Training set size is gradually increased for performance comparison, whereas validation and test sets are fixed

Dataset size (images)	Chronological age			Sex		
	MAE (years)	MD (years)	R^2^	ACC	SPE	SEN
2640	3.63 ± 2.98^*^	15.19	0.933	0.963^‡,**^	0.989	0.938
5280	3.48 ± 2.92^†^	17.18	0.938	0.978	0.973	0.982
7920	2.93 ± 2.61	16.31	0.957	0.992	0.993	0.990

MAE, mean absolute error; R^2^, coefficient of determination; ACC, accuracy; SPE, specificity; SEN, sensitivity. ^*^Significant difference for MAE between data sizes of 7920 and 2640 images (*p*-value < 0.05); ^†^Significant difference for MAE between data sizes of 7920 and 5280 images (*p*-value < 0.05); ^‡^Significant difference for sex estimation performance between data sizes of 7920 and 2640 images (*p*-value < 0.05); ^**^Significant difference for sex estimation performance between data sizes of 5280 and 2640 images (*p*-value < 0.05)

To interpret the decision-making processes of ForensicNet, Grad-CAM was used to visualize regions that contributed the most to the output decision (5). The heatmap regions generated by Grad-CAM from ForensicNet varied significantly, depending on the chronological age group. For patients younger than 30 years, ForensicNet focused on the near nasal bone, coronoid process, molar teeth, and their surrounding alveolar bone. In the panoramic radiographs of patients older than 40 years, regions near the upper and lower teeth, including dental implants, amalgam fillings, and dental crowns contributed more to estimating sex and chronological age.

## Discussion

Forensic dentistry uses dental evidence and parameters to identify individuals, reconstruct events, and assess the trauma. One of the most important applications of forensic dentistry is sex and chronological age estimation for human identification during mass disasters, homicides, and accidents [43]. Various dental-related parameters obtained from morphological measurements of anatomical structures, such as the maxillofacial bones, teeth, and frontal and paranasal sinuses, have been used in forensic dentistry to estimate sex and chronological age [8, 44, 45]. These anatomical structures were assessed using panoramic radiographs commonly used in the dental field, which provide a broad view of the maxillofacial region [11]. Recently, deep learning has been widely used in forensic dentistry to estimate sex and chronological age from panoramic radiographs [28, 29]. However, previous studies have used datasets with insufficient or non-uniform sex and age distributions, which could lead to inaccurate estimation for a particular sex or age owing to data bias. In this study, ForensicNet was proposed to simultaneously estimate sex and chronological age from panoramic radiographs. To mitigate bias in the data distribution, our dataset was built using 13,200 images with 100 images for each sex and age range from 15 to 80 years.

The estimation performance of backbones such as VGG16, MobileNet v2, ResNet10, DenseNet121, Vision Transformer, Swin Transformer, TransNet, and EfficientNet-B3 used in ForensicNet was compared. In our experiments, EfficientNet-B3 outperformed the other backbones in estimating both sex and chronological age from panoramic radiographs (Table 2). ForensicNet with EfficientNet-B3 achieved a superior performance owing to three key factors. EfficientNet-B3 utilizes a compound scaling method that simultaneously optimizes the depth, width, and resolution of the deep network [40]. This approach allows for better model representation and feature extraction across different scales and complexities of anatomical structures in panoramic radiographs. Second, sex and age attention branches were designed, including CBAM, which promoted a deep network to focus on anatomical features related to estimating sex and chronological age from panoramic radiographs. The proposed sex and age attention branches improved the estimation performance for both sex and chronological age, and their effectiveness was demonstrated by an ablation study, as shown in Table 5. In addition, ForensicNet demonstrated accurate and robust estimation of sex and chronological age in panoramic radiographs obtained from patients older than 50 years by learning anatomical context features using CBAM (Table 6). Finally, ForensicNet achieved superior performance by adopting a multi-task learning approach to simultaneously estimate sex and chronological age from panoramic radiographs. The primary reason for this improvement is that chronological age and sex are often correlated [46], and ForensicNet can learn complementary contextual information between sex and age using a multi-task learning approach.

We observed that ForensicNet with EfficientNet-B3 outperformed the other Transformer-based backbones for most evaluation metrics, particularly in the estimation performance of chronological age (Table 2). There are two factors that we believe contribute to the superior performance of EfficientNet-B3 over Transformer-based backbones: (1) In our task for chronological age and sex estimation, local patterns of anatomical structures are more important than global long-range relationships between anatomical structures. Because most previous works based on manual analysis focused on shapes and volumes of each local anatomical structure such as teeth [47], mandibular angle [18], maxillary sinuses [9], and pulp chamber [20, 48] to estimate chronological age and sex from panoramic radiographs. (2) Transformers, lacking inductive biases such as locality and translation invariance of CNNs, sometimes require substantially more datasets to learn the same local features and textures [49]. Although we collected 13,200 images with 100 images for each sex and age range of 15–80 years, the size of the dataset is not guaranteed to be sufficient to train the Transformer-based backbones.

ForensicNet exhibited a relatively higher estimation performance for chronological age in younger age groups than in older age groups (Table 4). The different developmental signs in the teeth during the growth and adolescent phases allow for more accurate age estimation for these individuals [50], where the tooth eruption sequence, tooth calcification, and root development are common tooth development indicators [13, 14]. The results from ForensicNet showed that the estimation performance of chronological age gradually decreased in the panoramic radiographs of patients aged over 50 years. Older patients typically undergo mechanical and chemical dental wear and dental treatments [51]. In addition, cumulative periodontal destruction of the alveolar bone owing to tooth decay is typically observed in older patients [52]. Furthermore, the teeth condition is highly diverse among older patients owing to socio-environmental factors such as education level, access to healthcare, and socioeconomic status [53, 54]. These factors further complicate chronological age estimation in panoramic radiographs of older patients [51]. The activation of the heatmap regions generated by Grad-CAM became more diverse and complex with increasing age, as shown in Fig. 5. ForensicNet achieved superior sex estimation performance (Table 2). As the influence of hormones, morphological shape and size differences are present between males and females in the maxillofacial bone and teeth [55], which allows for a relatively higher performance of sex estimation.

In Fig. 5, Grad-CAM was used to visualize the regions that contributed significantly to the decision regarding the output of ForensicNet. For sex and chronological age estimation, heatmap regions with high activation generated by Grad-CAM appeared on the nasal bone, mandible, second and third molars with their surrounding alveolar bone, and coronoid process area across all ages in panoramic radiographs (see Supplementary Materials for Figures S1-S6). In previous studies, the nasal bone was used as an indicator for assessing dental parameters such as nasal height, nasal width, and pyriform aperture for sex estimation [56]. The third molars and their surrounding alveolar bone show sexual dimorphism between males and females, and the third molars of males have more enamel deposition than those of females [57]. The shape of the coronoid process exhibits sexual dimorphism between males and females [58]. Molar teeth and their surrounding alveolar bone contain informative indicators for estimating chronological age from panoramic radiographs [47]. The pulp dimensions of the mandibular first molar are significant indicators of chronological age [48]. Pulp dimensions decrease with age owing to secondary dentin deposition, tooth mineralization, and dental attrition [6]. The accumulated changes in the alveolar bone resulting from periodontitis can be utilized as indicators for chronological age estimation [59]. On panoramic radiographs of older patients, complex activation of heatmap regions related to dental treatment, including dental prosthetics and implants. As depicted in Fig. 5, the activation regions generated by Grad-CAM from ForensicNet were similar to the anatomical regions used as indicators in previous studies on sex and chronological age estimation from panoramic radiographs.

ForensicNet was compared with previous studies based on deep learning for sex and chronological age estimation from panoramic radiographs (Table 9) [26–30]. In age estimation, Milošević et al. [26] reported an MAE of 3.96 on the dataset with a non-uniform age distribution between the younger and older age groups, while the proposed ForensicNet achieved an MAE of 2.93 ± 2.61 on the dataset with uniform age distribution ranging from 15 to 80 years. Bu et al. [27] obtained ACC and SEN values for sex estimation using 10,703 panoramic radiographs from samples aged 5–25 years. In contrast, our ForensicNet achieved values of 0.992 for ACC, 0.990 for SEN, and 0.993 for SPE, respectively. Two deep learning-based methods simultaneously estimated sex and age from panoramic radiographs. Vila–Blanco et al. [28] proposed DASNet for sex and age estimation on 2,289 panoramic radiographs acquired from patients aged 4.5 to 89.2 years. They reported an ACC of 0.854 for sex estimation and an MAE of 2.84 ± 3.75 for chronological age estimation. Similarly, Fan et al. [29] proposed a Transformer-based model for sex and chronological age estimation on 15,195 panoramic radiographs acquired from patients aged 16–50 and achieved an ACC of 0.955 for sex estimation and an MAE of 2.61 for chronological age estimation. Zhang et al. [30] proposed a sex-prior guided Transformer-based model for chronological age estimation on 10,703 panoramic radiographs acquired from patients aged 5–25 and achieved an MAE of 0.80 for chronological age estimation. However, previous studies evaluated their deep learning models on small test sets that had a relatively higher proportion of young females compared to older subjects. ForensicNet was evaluated on a test set with uniform sex and chronological age distribution from 15 to 80 years to minimize the impact of data bias and obtained a comparable estimation performance for sex and chronological age.

**Table 9:** Qualitative comparison with previous methods for sex and chronological age estimation from panoramic radiographs using deep learning

Methods	Dataset size	Age range	Data distribution	Chronological age	Sex		
				MAE (years)	ACC	SPE	SEN
Milošević et al. [26]	4,035	18–90	Non-uniform	3.96			
Bu et al. [27]	10,703	5–25	Non-uniform		0.867		0.867
Vila-Blanco et al. [28]	2,289	4.5–89.2	Non-uniform	2.84 ± 3.75	0.854		
Fan et al. [29]	15,195	16–50	Non-uniform	2.61	0.955		
Zhang et al. [30]	10,703	5–25	Non-uniform	0.80			
ForensicNet	13,200	15–80	Uniform	2.93 ± 2.61	0.992	0.993	0.990

MAE, mean absolute error; ACC, accuracy; SPE, specificity; SEN, sensitivity

**Fig. 8:**
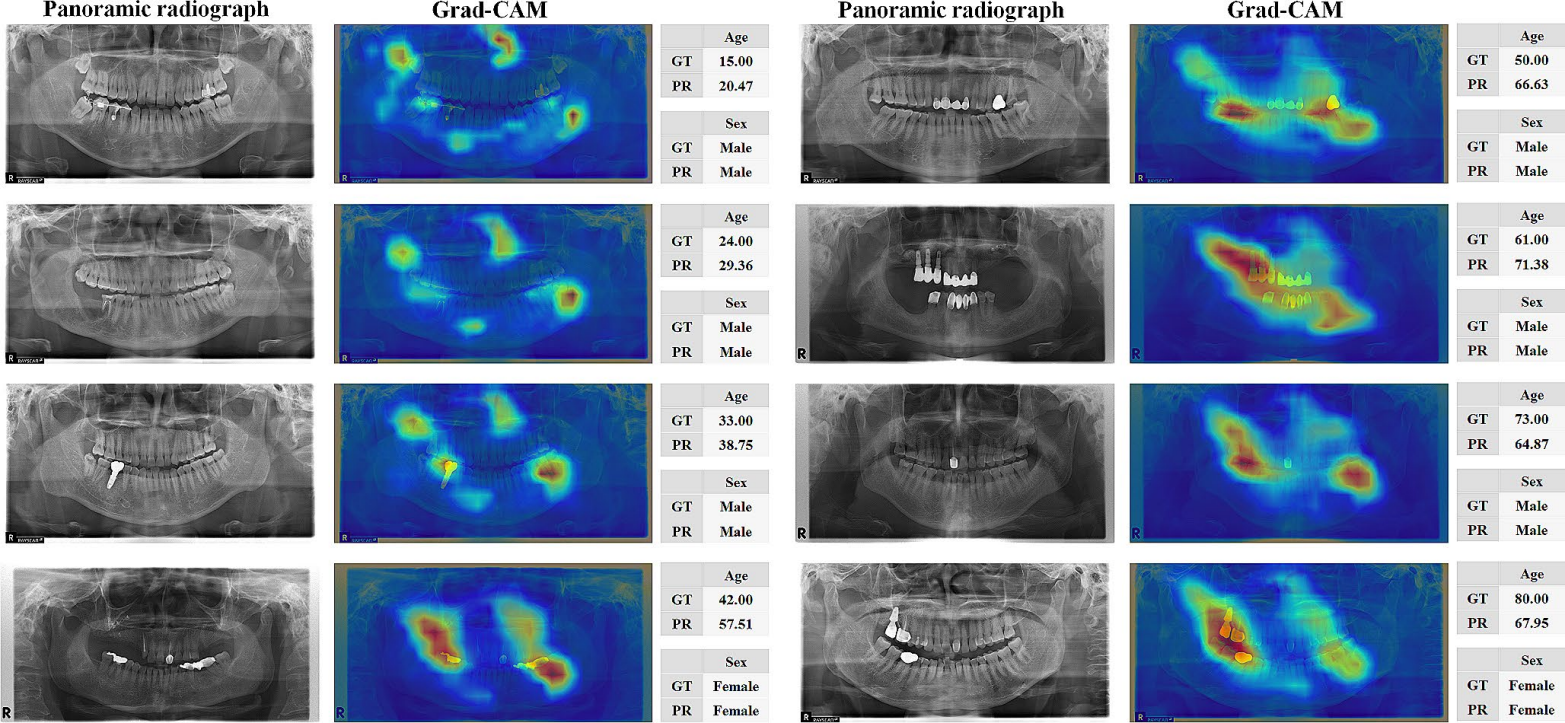
Representative estimation errors and corresponding Grad-CAM generated by EfficientNet-B3. GT and PR are the ground truth and estimation results, respectively

Estimation errors occurred in certain patients whose dental conditions differed from the typical dental conditions in that age group (Fig. 8). The chronological age estimated by ForensicNet was overestimated compared with the actual age of patients with tooth loss, dental treatment, or periodontitis for their age, with their regions activated by Grad-CAM on panoramic radiographs. Conversely, the estimated chronological age was underestimated compared to the actual age, particularly in patients who maintained excellent dental conditions and received minimal dental treatment for their age. Therefore, a lower chronological age estimation performance was observed in the panoramic radiographs of patients with significantly different dental conditions compared to those in the same age group.

Automatically estimating sex and chronological age using panoramic radiographs is difficult because of three major challenges. The first challenge is related to skeletal development and oral health conditions among patients, influenced by various factors such as age, sex, genetics, and environmental and oral health conditions. As adults undergo skeletal changes more slowly and are influenced by various factors, including genetics, lifestyle, and environmental conditions. Furthermore, accurate sex and chronological age estimation of elderly patients is generally more difficult than those of children owing to variations in dental conditions including dental implants, crowns, fillings, tooth caries, and missing teeth observed in elderly patients [6]. Therefore, estimating the sex and chronological age of adults may become more difficult than that of children [53]. Second, panoramic radiographs have overlapping anatomical structures and various imaging positions, contrasts, and resolutions [60], making it difficult to estimate sex and chronological age. Clinical practice requires an automated method that is accurate and robust against variations in image quality and the presence of overlapping anatomical structures. The latter is related to data collection bias, such as unbalanced data distribution across different sex and age groups. When the data are unbalanced, a deep learning network may learn to focus on the majority class and overlook the minority class [61]. This may lead to inaccurate estimations for the minority class.

The following issues will be addressed in future studies to improve the estimation performance of ForensicNet. First, our dataset was built using panoramic radiographs from patients aged 15 to 80 years, all of whom had nearly finished developing their permanent dentition and maxillofacial bone growth. Additional datasets from children and adolescents with mixed dentition or incomplete mandibular growth are required to improve the capability of our method for sex and chronological age estimation. Second, our method may have limited generalizability. It relies solely on internal data including only living individuals from a single organization in South Korea, which might not be representative of deceased individuals, broader populations, or different organizational contexts. Therefore, further research is required to train and evaluate ForensicNet using panoramic radiograph datasets collected by multiple organizations and devices from deceased individuals, diverse ethnicities, and populations. Finally, several exclusion criteria were set for collecting panoramic radiographs. In future studies, we will improve the generalizability and clinical efficacy of ForensicNet using large-scale panoramic radiographs of all ages, including the excluded samples. In addition, we plan to study an optimal hybrid model of Transformer, CNN, and Diffusion models to improve estimation performance for the chronological age and sex of ForensicNet [30, 49, 62].

## Conclusion

In this study, an automatic and robust network (ForensicNet) was proposed for both sex and chronological age estimation from panoramic radiographs. The network was trained and evaluated using a large dataset with a uniform distribution of sex and age ranging from 15 to 80 years. ForensicNet with EfficientNet-B3 outperformed the other backbones in estimating sex and chronological age and demonstrated accurate and robust estimation of sex and chronological age from panoramic radiographs for patients older than 50 years by learning anatomical context features using the proposed sex and age attention branches with CBAM. This method is expected to enable the automatic and robust estimation of sex and chronological age and improve the workflow of forensic investigation and research for individual identification. In future studies, we will improve the generalizability and clinical efficacy of ForensicNet using large-scale panoramic radiographs collected by multiple organizations and devices from diverse ethnicities and populations.

### Electronic supplementary material

Below is the link to the electronic supplementary material.

Supplementary Material 1

Supplementary Material 2

Supplementary Material 3

Supplementary Material 4

Supplementary Material 5

Supplementary Material 6

Supplementary Material 7
