# Supplementary material for: Automatic and robust estimation of sex and chronological age from panoramic radiographs using a multi-task deep learning network: a study on a South Korean population
Source: Int J Legal Med. 2024 Mar 12;138(4):1741–57. doi: 10.1007/s00414-024-03204-4 (PMC11164743; doi:10.1007/s00414-024-03204-4)
Supplement: Supplementary file 1 — Supplementary Material 1 [file 414_2024_3204_MOESM1_ESM.docx]

**Supplementary Material**

**Automatic and robust estimation of** **sex and chronological age from panoramic radiographs using a multi-task deep learning network: A study on a South Korean population**

*Sejin Park^1,*^, Su Yang^2,*^, Jun-Min Kim^3^, Ju-Hee Kang^1^, Jo-Eun Kim^1^, Kyung-Hoe Huh^1^, Sam-Sun Lee^1^, Won-Jin Yi^1,2,^****^†^****, Min-Suk Heo^1,^****^†^****,*

*^1^Department of Oral and Maxillofacial Radiology and Dental Research Institute, School of Dentistry, Seoul National University, Seoul, 03080 South Korea*

*^2^Department of Applied Bioengineering, Graduate School of Convergence Science and Technology, Seoul National University, Seoul, 03080 South Korea*

*^3^Department of Electronics and Information Engineering, Hansung University, Seoul, 03080 South Korea*

*^*^These authors contributed equally*

***^†^****These authors are co-corresponding authors*

**Corresponding author**

Department of Oral and Maxillofacial Radiology, School of Dentistry, Seoul National University, 101

Daehak-ro, Jongno-gu, Seoul 110-768, Korea

| *Min-Suk Heo, DDS*  *Tel: +82-2-6256-3051*  *E-mail:* [*hmslsh@snu.ac.kr*](mailto:hmslsh@snu.ac.kr) | *Won-Jin Yi, Ph.D*  *Tel: +82-2-6256-3052*  *E-mail: wjyi@snu.ac.kr* |
| --- | --- |

**Acknowledgements**

This study was supported by Grant No. 02-2022-0220 from the SNUDH Research Fund and the National Research Foundation of Korea (NRF) Grant funded by the Korean Government (MSIT) (No. 2023R1A2C200532611). This study was also supported by a Korea Medical Device Development Fund Grant by the Korean government (Ministry of Science and ICT; Ministry of Trade, Industry, and Energy; Ministry of Health and Welfare; Ministry of Food and Drug Safety) (Project Number: 1711194231, KMDF_PR_20200901_0011, 1711174552, KMDF_PR_20200901_0147).

In this **supplementary information**, we provide average panoramic radiographs with corresponding Grad-CAM on each age group and additional estimation results with corresponding Grad-CAM generated by EfficientNet-B3. Next, we supply discussions on the results of average Grad-CAM on each age group.

In Figure S1, we observed average heatmap regions with high activation generated by Grad-CAM appearing near the nasal bone, mandible, second and third molars with their surrounding alveolar bone, and coronoid process area across all ages in panoramic radiographs. On panoramic radiographs of older patients, complex activation of average heatmap regions spread near teeth including dental prosthetics and implants. In Figures S2-S6, heatmap regions generated by Grad-CAM from ForensicNet were also activated at similar regions depicted in Figure S1 for each age group. Consequently, the average activation regions generated by Grad-CAM from ForensicNet were similar to the anatomical regions used as indicators in previous studies on sex and chronological age estimation from panoramic radiographs.


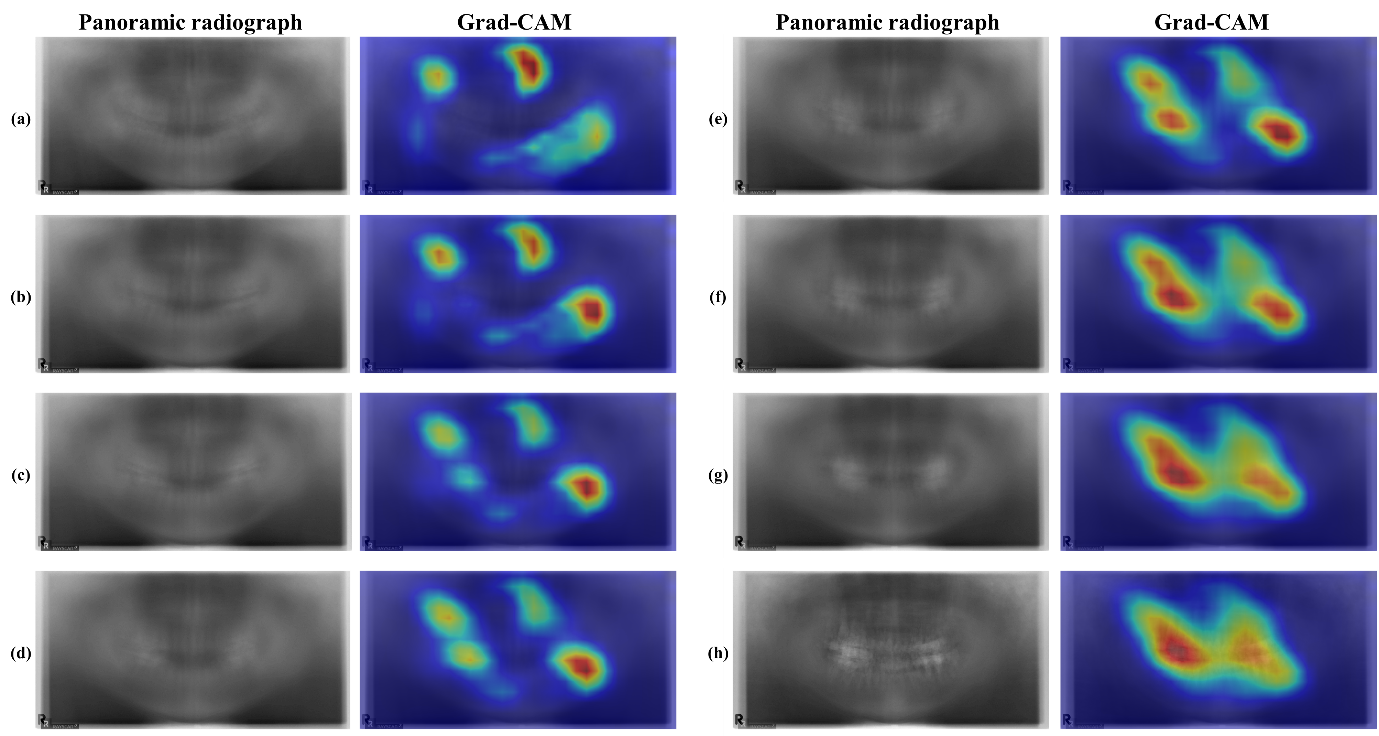


**Fig S1.** Average panoramic radiographs and corresponding Grad-CAM generated by EfficientNet-B3. (a)–(h) Results on each age group for [15–20), [20–30), [30–40), [40–50), [50–60), [60–70), and [70–80], respectively.


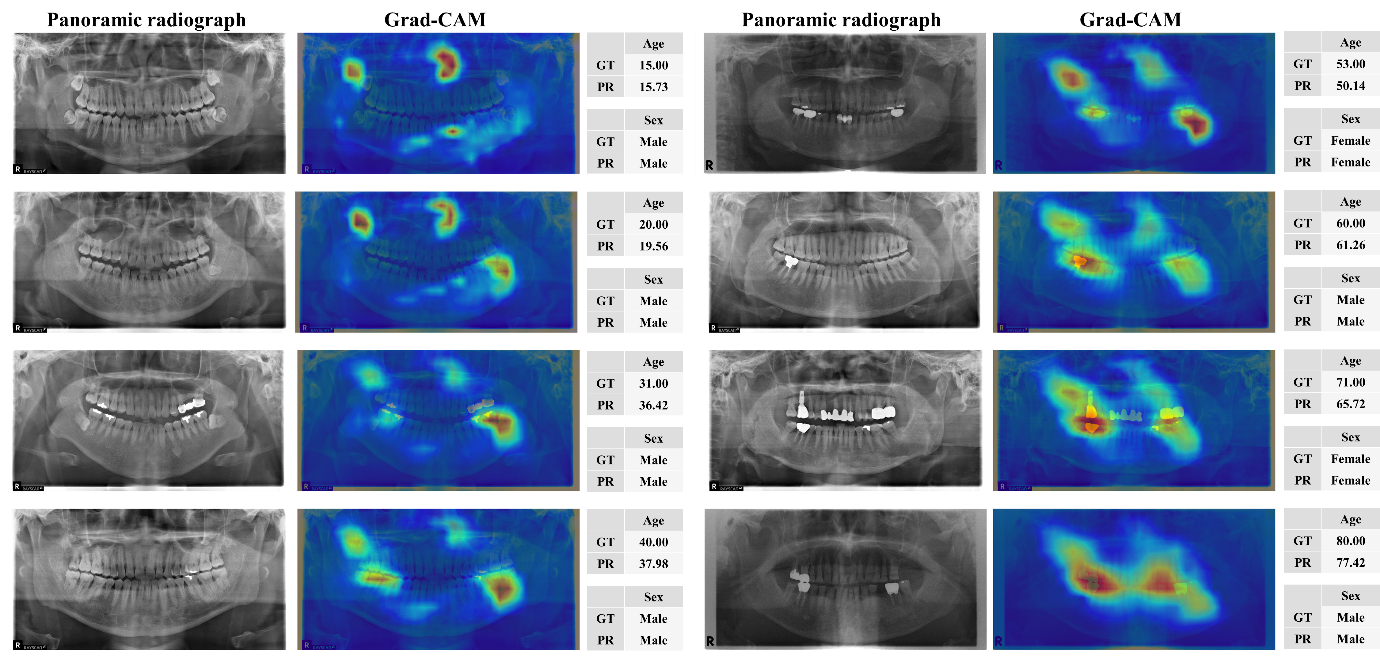


**Fig. S2** Representative estimation errors and corresponding Grad-CAM generated by EfficientNet-B3. GT and PR are the ground truth and estimation results, respectively


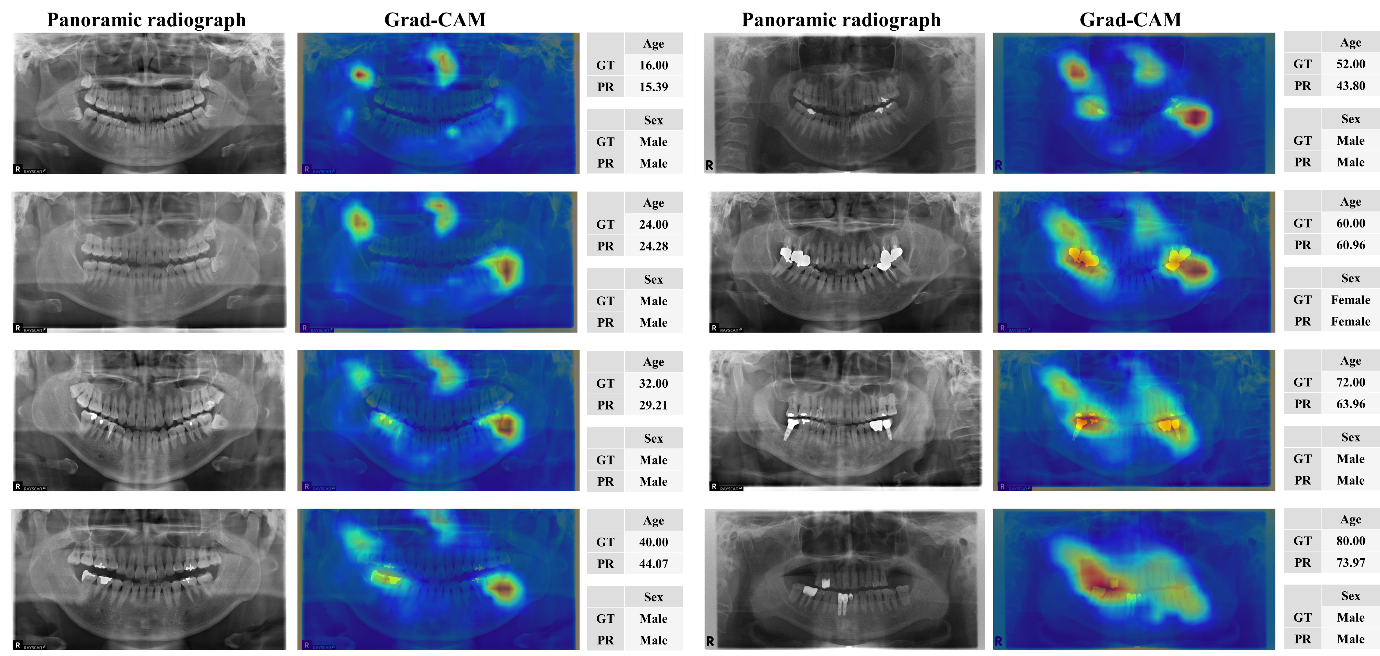


**Fig. S3** Representative estimation errors and corresponding Grad-CAM generated by EfficientNet-B3. GT and PR are the ground truth and estimation results, respectively


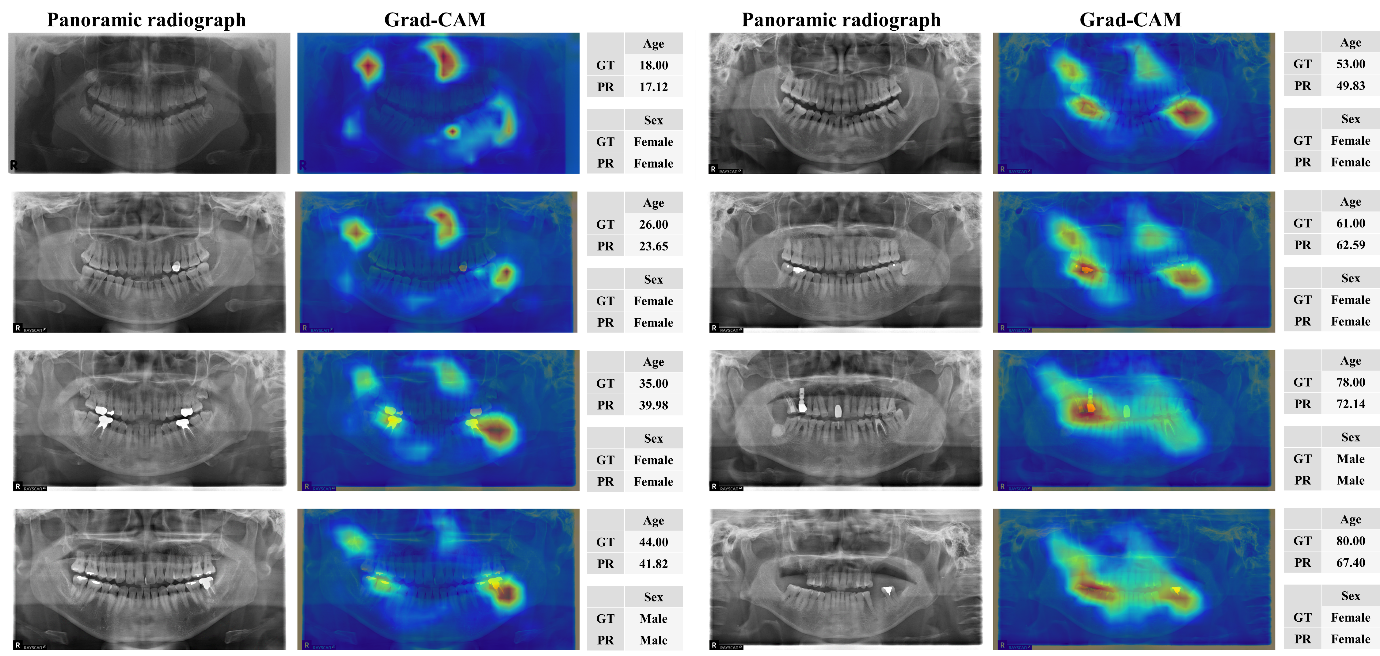


**Fig. S4** Representative estimation errors and corresponding Grad-CAM generated by EfficientNet-B3. GT and PR are the ground truth and estimation results, respectively


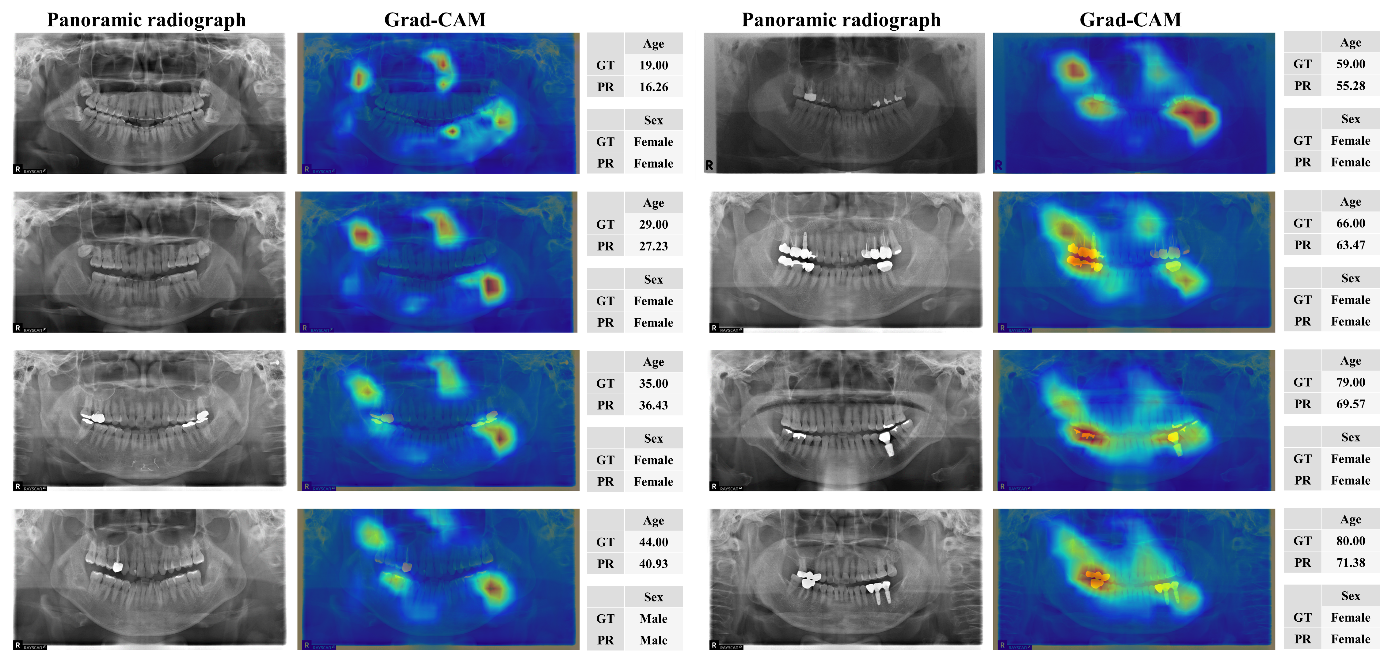


**Fig. S5** Representative estimation errors and corresponding Grad-CAM generated by EfficientNet-B3. GT and PR are the ground truth and estimation results, respectively


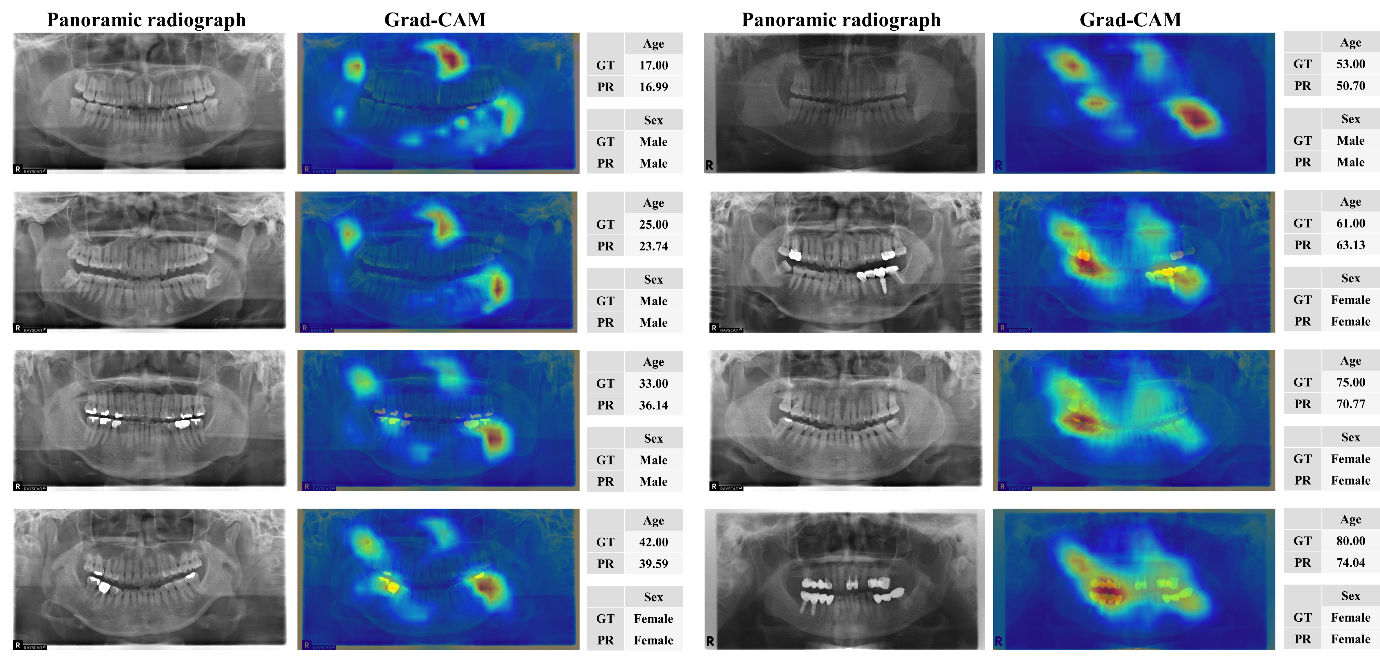


**Fig. S6** Representative estimation errors and corresponding Grad-CAM generated by EfficientNet-B3. GT and PR are the ground truth and estimation results, respectively
