# Supplementary figures and images for: Automatic and robust estimation of sex and chronological age from panoramic radiographs using a multi-task deep learning network: a study on a South Korean population
Source: Int J Legal Med. 2024 Mar 12;138(4):1741–57. doi: 10.1007/s00414-024-03204-4 (PMC11164743; doi:10.1007/s00414-024-03204-4)

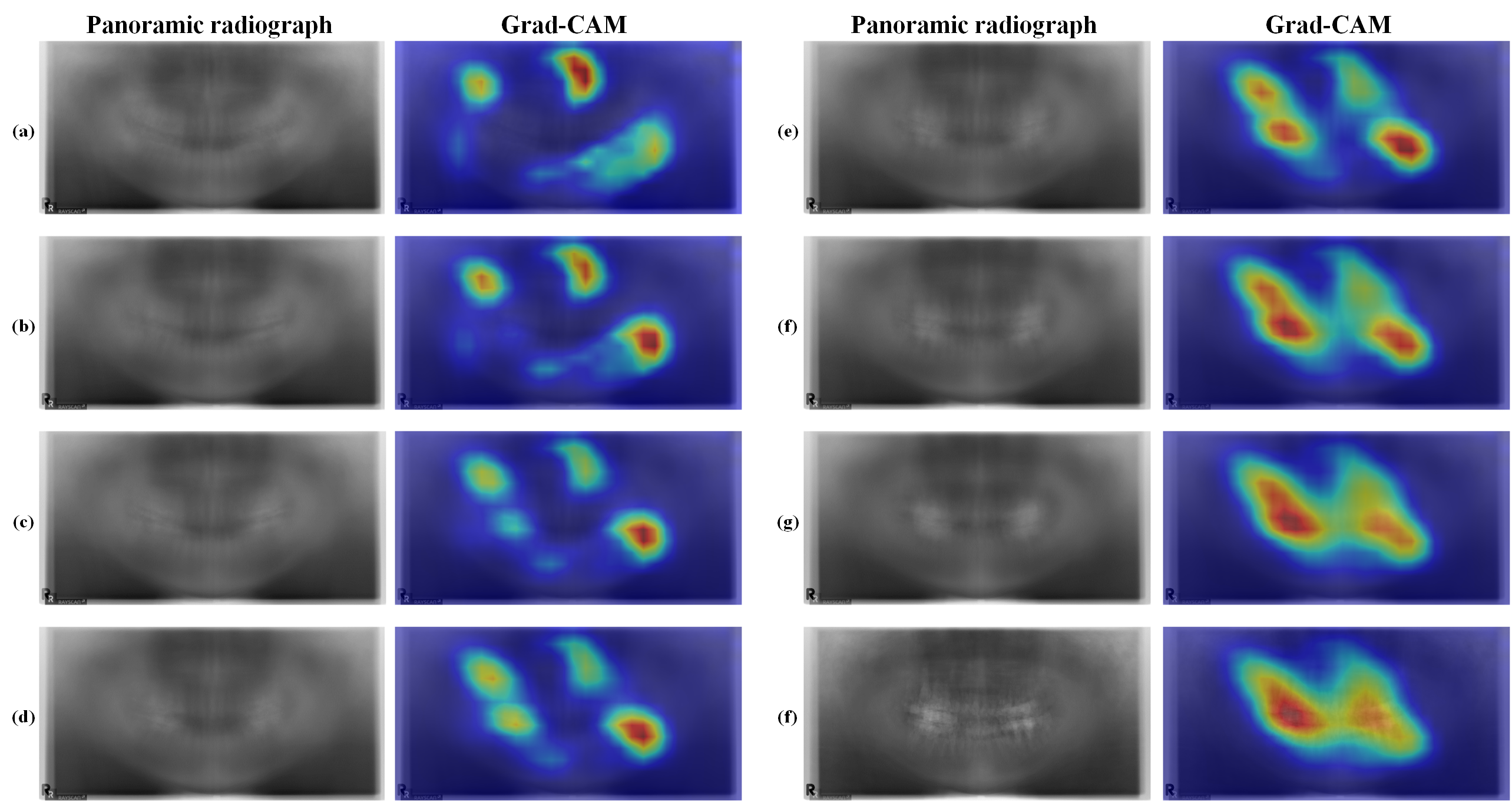

Supplement: Supplementary file 2 — Supplementary Material 2 [file 414_2024_3204_MOESM2_ESM.png]

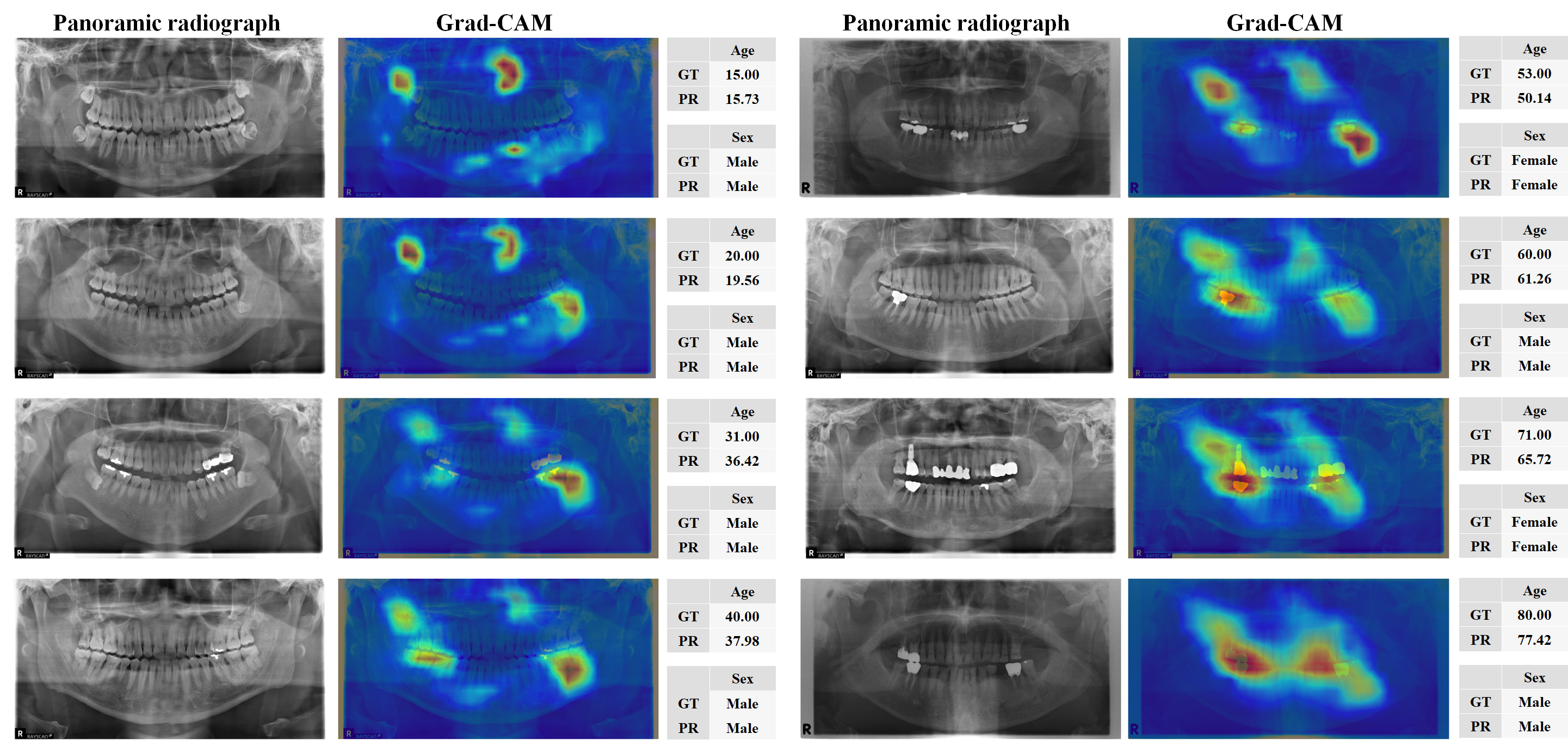

Supplement: Supplementary file 3 — Supplementary Material 3 [file 414_2024_3204_MOESM3_ESM.png]

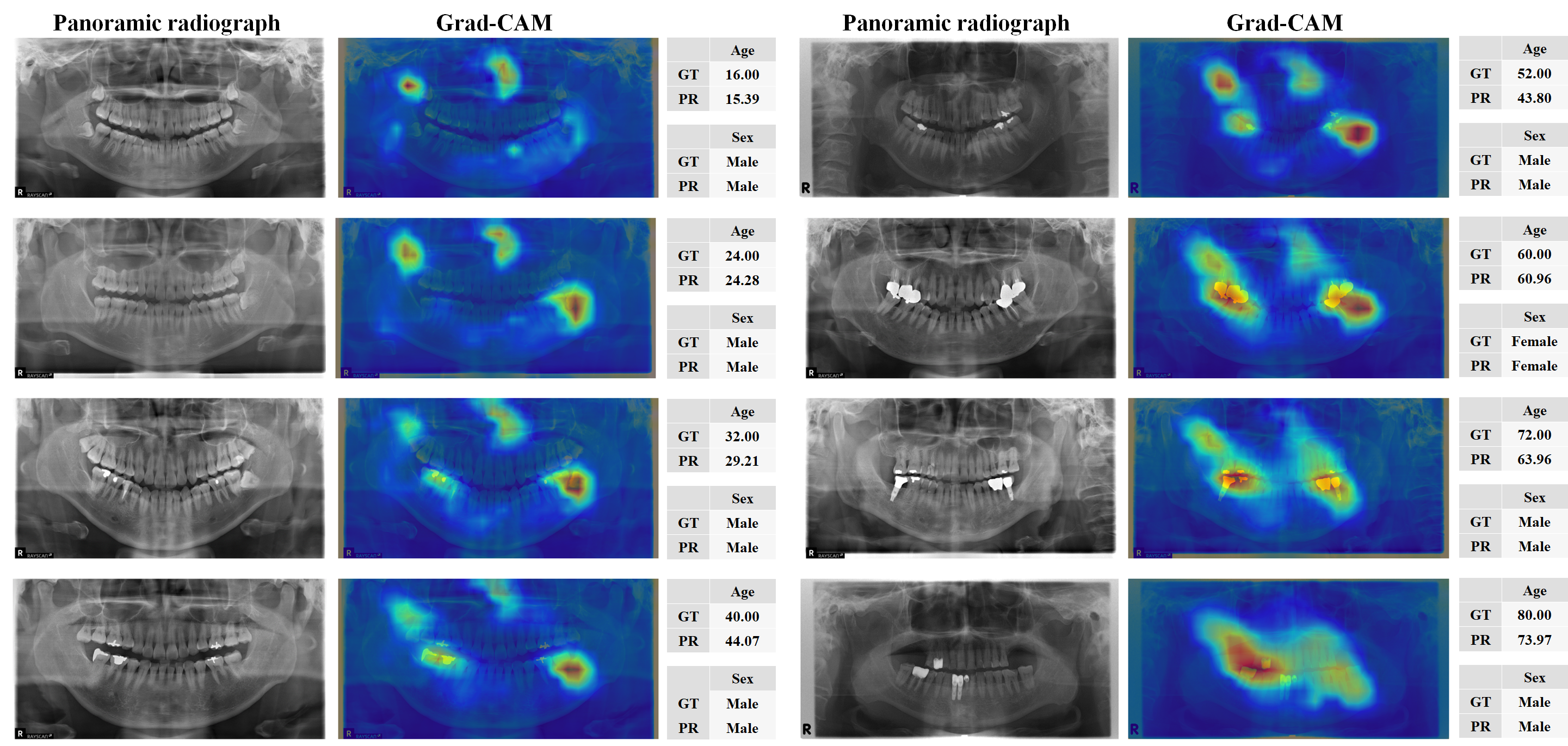

Supplement: Supplementary file 4 — Supplementary Material 4 [file 414_2024_3204_MOESM4_ESM.png]

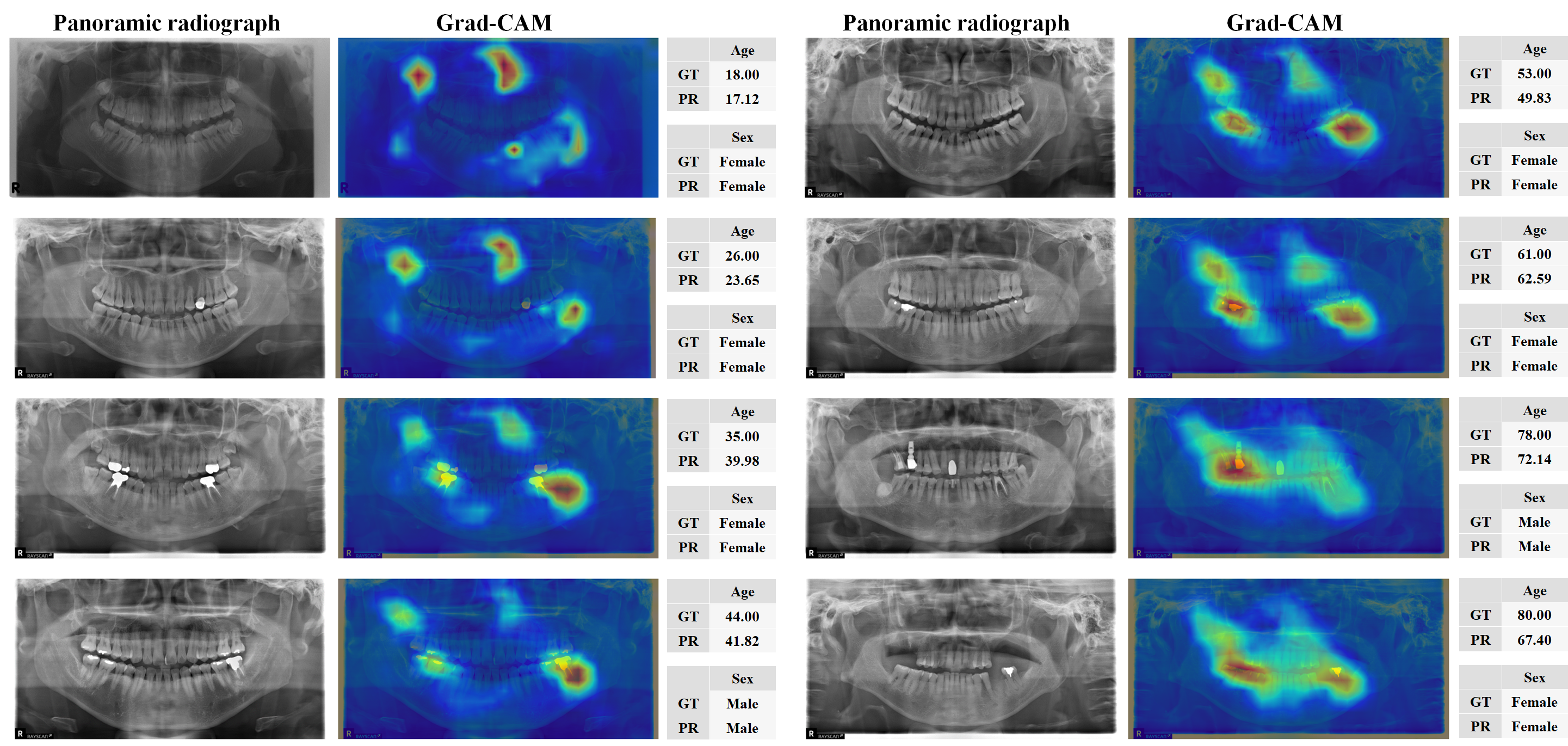

Supplement: Supplementary file 5 — Supplementary Material 5 [file 414_2024_3204_MOESM5_ESM.png]

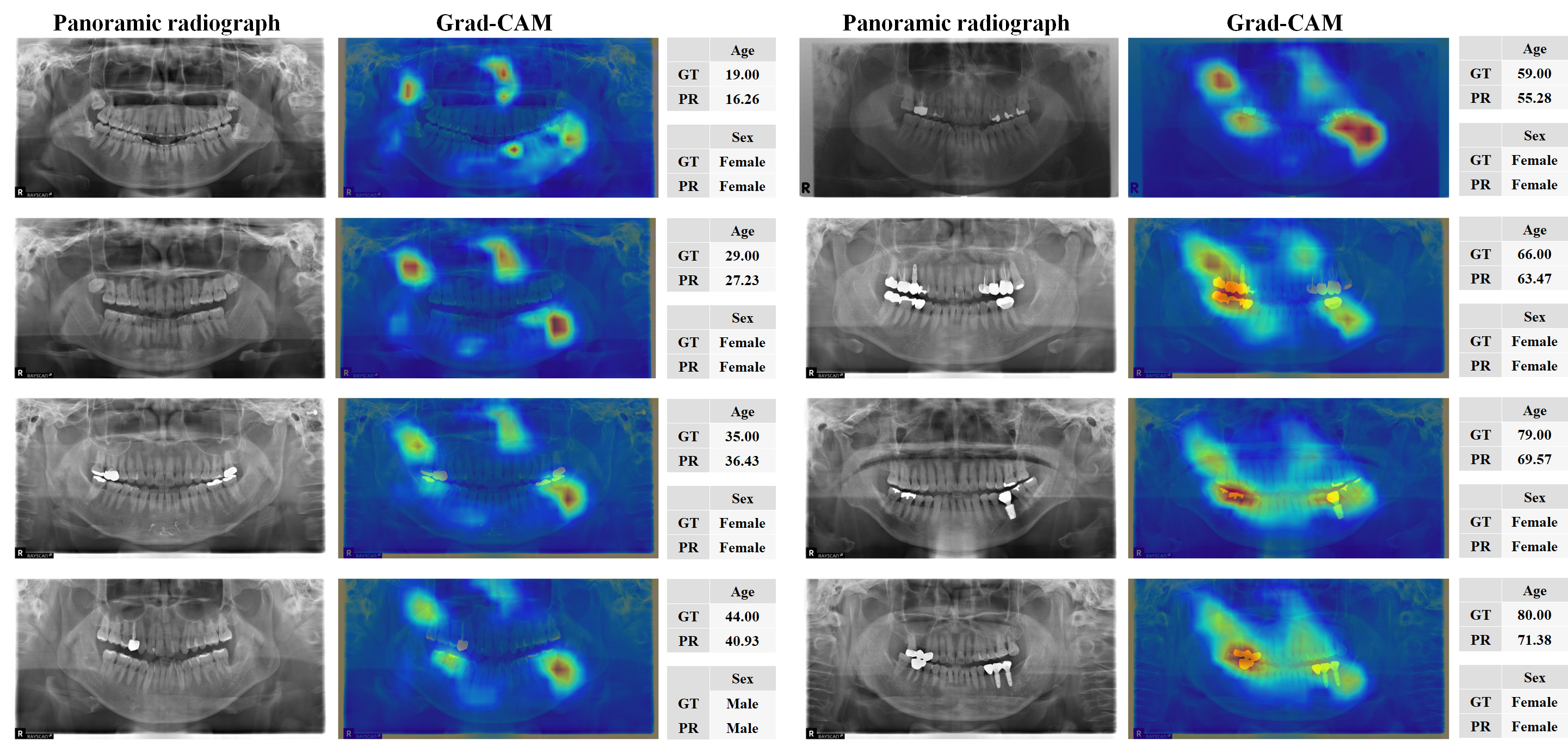

Supplement: Supplementary file 6 — Supplementary Material 6 [file 414_2024_3204_MOESM6_ESM.png]

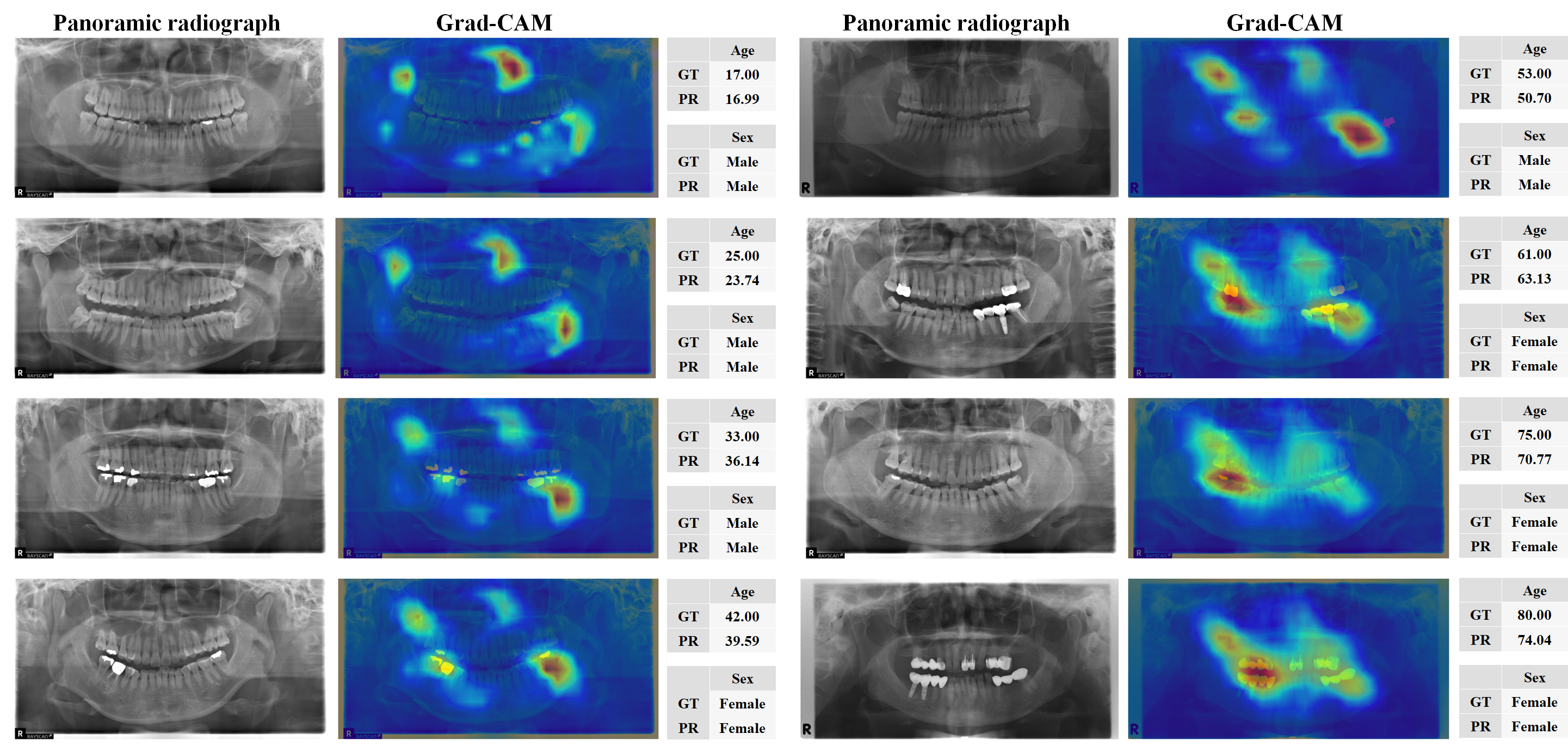

Supplement: Supplementary file 7 — Supplementary Material 7 [file 414_2024_3204_MOESM7_ESM.png]
